# The *qTSN* Positive Effect on Panicle and Flag Leaf Size of Rice is Associated with an Early Down-Regulation of Tillering

**DOI:** 10.3389/fpls.2015.01197

**Published:** 2016-01-07

**Authors:** Dewi E. Adriani, Tanguy Lafarge, Audrey Dardou, Aubrey Fabro, Anne Clément-Vidal, Sudirman Yahya, Michael Dingkuhn, Delphine Luquet

**Affiliations:** ^1^CIRAD, UMR AGAP, F-34398 MontpellierFrance; ^2^Faculty of Agriculture, University of Lambung MangkuratBanjarbaru, Indonesia; ^3^Crop and Environment Science Division, International Rice Research InstituteLos Baños, Philippines; ^4^Department of Agronomy and Horticulture, Bogor Agricultural UniversityBogor, Indonesia

**Keywords:** rice, *qTSN4*, down-regulation of tillering, panicle size, top leaf and internode size, main stem growth rate

## Abstract

The *qTSN4* was identified as rice QTL (Quantitative Traits Locus) increasing total spikelet number per panicle and flag leaf area but potentially reducing panicle number depending on the environment. So far, this trade-off was mainly observed at grain maturity and not specifically studied in details, limiting the apprehension of the agronomic interest of *qTSN4*. This study aimed to understand the effect of *qTSN4* and of the environment on panicle sizing, its trade-off with panicle number, and finally plant grain production. It compared two high yielding genotypes to their Near Isogenic Lines (NIL) carrying either QTL *qTSN4* or *qTSN12, two* distinct QTLs contributing to the enlarged panicle size, thereafter designated as *qTSN*. Traits describing C sink (organ appearance rate, size, biomass) and source (leaf area, photosynthesis, sugar availability) were dynamically characterized along plant and/or panicle development within two trials (greenhouse, field), each comparing two treatments contrasting for plant access to light (with or without shading, high or low planting densities). The positive effect of *qTSN* on panicle size and flag leaf area of the main tiller was confirmed. More precisely, it could be shown that *qTSN* increased leaf area and internode cross-section, and in some cases of the photosynthetic rate and starch reserves, of the top 3–4 phytomers of the main tiller. This was accompanied by an earlier tillering cessation, that coincided with the initiation of these phytomers, and an enhanced panicle size on the main tiller. Plant leaf area at flowering was not affected by *qTSN* but fertile tiller number was reduced to an extent that depended on the environment. Accordingly, plant grain production was enhanced by *qTSN* only under shading in the greenhouse experiment, where panicle number was not affected and photosynthesis and starch storage in internodes was enhanced. The effect of *qTSN* on rice phenotype was thus expressed before panicle initiation (PI). Whether early tillering reduction or organ oversizing at meristem level is affected first cannot be entirely unraveled. Further studies are needed to better understand any signal involved in this early regulation and the *qTSN* × Environment interactions underlying its agronomic interest.

## Introduction

Grain yield elaboration in cereals depends on the establishment of panicle number per square meter and panicle size, grain filling rate, and individual grain size (Chen et al., 2008; Gaju et al., 2014). All these traits are known to compensate per unit area i.e., among plants (Zhang and Yamagishi, 2010) and within the plant where they compete for the same pool of C and N resources (Okawa et al., 2003; Hashida et al., 2013). Amongst these traits in rice, panicle number, and panicle size are those characterized with the highest plasticity under favorable growing conditions thus with the highest impact on yield elaboration. These two traits are determined along plant cycle, particularly based on tillering and green leaf area dynamics, internode reserve remobilization, and reproductive sink size and number. All these processes are depending on C assimilated and N availability, as reported by Lafarge and Bueno (2009) and Dingkuhn et al. (2015) in rice, and by Dreccer et al. (2008) in wheat. Regarding panicle size, many studies reported that panicle development, and the determination of spikelet number per panicle, is closely correlated to early plant vigor and underlying traits such as leaf appearance rate (Dong et al., 2004; Streck et al., 2009; Itoh and Shimizu, 2012; Rebolledo et al., 2012), tillering (Lafarge et al., 2002, 2010; Borràs-Gelonch et al., 2012), culm (Fujita and Yoshida, 1984; Wu et al., 2011), and peduncle (Liu et al., 2008) size, plant height (Wang et al., 2014; Chen et al., 2015), or even neck internode diameter (Zhang and Yamagishi, 2010).

Several genes were reported to control morphogenetic processes positively during both the vegetative and the reproductive phases. Amongst them, the genes *MOC1* (Monoculm 1, Li et al., 2003) and LAX (Komatsu et al., 2003) were shown to promote axillary meristem initiation and accordingly to regulate both shoot and panicle branching. The *Gn1* gene is involved in the control of plant height and grain number per panicle (Ashikari et al., 2005). The *DEP1* enhances shoot apical meristem activity and grain number per panicle (Huang et al., 2009). The *OsSPL14* promotes early tillering and grain number per panicle (Miura et al., 2010). The *APO1* increases grain number per plant and harvest index (Ikeda et al., 2007; Terao et al., 2010). The *OsEBS* enhances plant biomass and spikelet number per panicle (Dong et al., 2013). By contrast, other genes were shown to have trade-off effects on some morphogenetic traits expressed during the vegetative and the reproductive phases. This is the case of the rice mutant *this1* characterized by higher tiller number but lower plant height and fertile spikelet number compared to the wild type (Liu et al., 2013). The *Ghd7* increases spikelet number per panicle through panicle branching but decreases tillering in a density-dependent manner (Weng et al., 2014); the OsSPS1 gene inhibits plant height but enhances spikelet number per panicle (Hashida et al., 2013). The *Ltn* (low tiller number) and similarly *SPIKE* (*qTSN4*) induces higher total spikelet number per panicle but decreases final panicle number (Fujita et al., 2010, 2013).

These studies point out that a gene has unlikely a positive effect on both organ size and number and accordingly that molecular breeding efforts are frequently confronted to the issue of physiological trade-offs among traits, compromising the direct use of a candidate gene or QTL in crop improvement. The trade-off between tiller number and organ size is of major concern for rice, not only with respect to vegetative growth (Rebolledo et al., 2012) but also reproductive growth as it comes as a parameter regulating panicle size vs. number (Lafarge et al., 2010). The *qTSN4* was recently identified as a QTL enhancing flag leaf width and total spikelet number per panicle in IR64 background (Fujita et al., 2009, 2012, 2013). This QTL is known to co-locate with *Nal1* gene involved in the determination of leaf structure, veining pattern, and carboxylation (Qi et al., 2008), as well as other early traits like stem length and vascular bundle number (Fujita et al., 2012, 2013). It was also shown that its positive effect on panicle size was potentially depressive on panicle number (Fujita et al., 2012, 2013). Recently, Okami et al. (2015) confirmed that an isogenic line carrying *qTSN4* produced fewer but larger tillers than its parent (IR64).

No studies, however, were conducted to further understand at plant level the physiological and environmental determinisms regulating (i) the trade-off between panicle size and number in genotypes introgressed with *qTSN4* and (ii) accordingly, the positive effect of *qTSN4* on plant grain production. This is the aim of the present study that, analyzes the effect of *qTSN* on plant growth and development and thus on yield formation processes along plant cycle in contrasting situations of plant access to light. Two high yielding genotypes (IR64 and IRRI146) were compared to their Near Isogenic Lines (NIL) carrying QTL *qTSN4* (Fujita et al., 2013) and QTL *qTSN12*, a different QTL reported for having a similar function as *qTSN4*, i.e., enhanced panicle size (Ishimaru, personal communication). This was conducted within three trials under contrasted situations of plant access to light and thus to C assimilates in order to modulate the level of competition among sinks within the plant. Plant growth was then characterized in terms of morphogenesis and biomass accumulation per organ type along plant development.

## Materials and methods

### Plant materials

Two pairs of parents/near-isogenic lines (P/NIL): IR64 vs. IR64 NIL and IRRI146 vs. IRRI146 NIL were studied in two experiments. In one of these experiments, an additional pair of P/NIL was considered asIR64 and NIL1 carrying *qTSN12*. This QTL has been observed to increase panicle size and reduce panicle number—as observed with *qTSN4*—and was detected in chromosome 12 from donor parent YP4. The NILs were developed by self-pollination of a plant selected from BC4F2 population (in IR64 background) and BC3F1 population (in IRRI146 background; Fujita et al., 2013). The IR64 NIL was identified by composite interval mapping carrying the high total spikelet number (TSN) QTL between Simple Sequence Repeat (SSR) markers RM3423 and RM17492 on the long arm of chromosome 4 (Fujita et al., 2012), whereas IRRI146 NIL was developed from recurrent backcrossing to IRRI146 and marker-assisted selection (MAS; Fujita et al., 2013). The details (cross combination, donor and category) of plant materials are available in Table 1. The recipient lines (parents) were chosen because of the wide adaptability as of IR64 as a mega variety grown in many parts of the world (Khush, 1987) and IRRI146, a 2nd-generation New Plant Type (NPT) variety developed at IRRI and released in the Philippines in 2007 under the name NSIC Rc158, a high-yielding *indica* cultivar as well (Brennan and Malabayabas, 2011).

**Table 1:** **Details of plant materials**.

**Designation**	**Species**	**Cross combination**	**Donor**	**Category**
IR64	Indica	IR64		Recurrent parent recipient
IR64 NIL	Isoline of IR64	(IR64/IR68522-10-2-2//3^*^IR64)/IR64	IR68522-10-2-2	IR64-qTSN4.4
IR64 NIL1	Isoline of IR64	(IR64/IR65564-2-2-3//3^*^IR64)/IR65	IR65564-2-2-3	IR64-qTSN12.2
IRRI146	Indica	NSIC Rc158		Recurrent parent recipient
IRRI146 NIL	Isoline of NSIC Rc158	NSIC Rc158/IR65564-2-2-3//3^*^NSIC Rc158	IR65564-2-2-3	NSIC Rc158-qTSN4.1

### Experiments

#### GH-CNRS

Experiment I was conducted in the greenhouse from May to August 2013 at the National Center for Scientific Research (CNRS), Montpellier, France. This experiment tested *qTSN*4 in IRRI146 and IR64 genetic backgrounds comparing one control treatment (C) and a shading (S) treatment with a 58% light attenuation from panicle initiation (PI) up to heading (H) by using a gray net all around the table bringing the plants.

The seeds were grown in a germination chamber at 29°C, then transplanted 4 days after germination in 3 l pots (three seeds per pot) when seedlings were about 3 cm tall. The thinning of plant population to one plant per pot (downsizing to 45.65 plants m^−2^) was conducted at four-leaf stage. Pots contained about of their volume with EGOT 140 media (17N-10P-14K, pH of 5) and were placed side by side (corresponding to 14.8- cm spacing) on tables filled with 5 cm water depth. Basal fertilizer was applied using Basacot 6M+ at 2g l^−1^, 11N-9P-19K +2Mg incorporated before transplanting. Pots were arranged in four aluminum tables and each table was containing 104 pots, including border plants, at the beginning of the experiment. The tables were moved every week from 2 weeks after transplanting until maturity to avoid any bias due to the green house structure.

Weather data were collected from the AWS (*Automatic Weather Station*) that was installed in the center of the tables measuring Photosynthetic Active Radiation (PAR), global radiation (Rg), air temperature (T), and relative humidity (RH). The average daily air temperature throughout the crop cycle was 27.3 ± 0.6°C. The average daily PAR for the whole crop cycle was 24.7 ± 7.1 mol m^−2^ d^−1^ under full light and 10.3 ± 3.4 mol m^−2^ d^−1^ under shading, and the average RH was 66.8 ± 7.7%.

#### Field-IRRI

Experiment II was performed in the field at International Rice Research Institute (IRRI) experiment station in Los Baños, Philippines (14°11′N, 121°15′E, 21 m altitude), from December 2013 to April 2014. This experiment tested *qTSN4* and *qTSN12* effect in IRRI146 and IR64 genetic backgrounds and at two planting densities, low (LD, 25 plants m^−2^), and high density (HD, 100 plants m^−2^).

The seeds were soaked for 24 h, drained and incubated for another 24 h, then sown in the seeding trays in the greenhouse on December 5, 2013. The 2-week old seedlings were transplanted in the field at one plant per hill in a 2 × 2.4 m^2^ plots. The field was initially flooded to hold two puddlings and two harrowings, standing water level, 3–5 cm, was maintained as the IRRI guide field standard. Phosphorus (30 kg P ha^−1^), potassium (40 kg K ha^−1^), and zinc (5 kg Zn ha^−1^) were applied and incorporated into all the plots 2 days before transplanting. 60 kg N ha^−1^ was applied 1 day before transplanting, then 40 kg N ha^−1^ and 60 kg N ha^−1^ were applied at mid-tillering and PI stage, respectively.

Weather data were collected from the IRRI meteorological station measuring radiation (MJ m^−2^), daylight (h), rainfall (mm), evaporation (mm), average temperature (°C), vapor pressure (kPa), RH (%), and wind speed (m s^−1^). Average daily air temperature throughout crop cycle was 25.6 ± 1.5°C. The average daily PAR for the whole crop cycle was 31.0 ± 11.3 mol m^−2^ d^−1^, and the average RH was 84.2 ± 4.8%.

#### GH-IRRI

Experiment III was performed in an open-top green house during the wet season (August to November 2014) supporting the two experiments described above. This experiment adopted split plot design with three replications and was conducted at IRRI, Los Baños, Philippines (14°11′N, 121°15′E, 21 m altitude). The main factor was plant spacing: crowded density, 20 × 20 cm (Cr, 25 plants m^−2^) and isolated density, 60 × 60 cm (Is, 2.78 plants m^−2^) from PI up to flowering (FLO). The subsidiary factor was a pair of rice genotype: IRRI146 (NSIC Rc158) recipient line and its NIL (*qTSN4.1*–YP4).

The seeds were soaked for 24 h, drained and incubated for another 24 h, then sown in the 6 l pots (four seeds per pot). Pots contain about of its volume of Andaqueptic Haplaquoll with a topsoil of 39% clay, 46% silt, 14% sand, pH of 6.38. The thinning of plant population to one plant per pot was conducted 2 weeks after sowing. Water level in the pots was always maintained about 8 cm height. 4 g of Ammonium Sulfate (NH_4_)_2_(SO_4_), 2 g of Single Super Phosphate (SSP) and 2 g of muriate of Potash (KCl) were applied and incorporated into all the pots 2 days before sowing, and 2 g of Ammonium Sulfate was applied at PI stage.

Weather data were collected from IRRI meteorological station measuring radiation (MJ m^−2^), daylight (h), rainfall (mm), evaporation (mm), average temperature (°C), vapor pressure (kPa), RH (%) and wind speed (m s^−1^). Average daily air temperature throughout crop cycle was 27.7 ± 0.9°C. The average daily PAR for whole crop cycle was 29.8 ± 11.5 molm^−2^d^−1^, and RH was 85.7 ± 5.3%.

### Plant measurements

#### Plant development and biomass accumulation

In both GH-CNRS and field-IRRI, leaf appearance on the main tiller and green tiller number were measured every week from 2 weeks after sowing (in GH-CNRS) and 2 weeks after transplanting (in the field) up to flag leaf stage on 3 and 4 sampled plants in GH-CNRS and field, respectively, in every replication. Thermal time was calculated by daily integration of air temperature minus a base temperature of 12°C (Rebolledo et al., 2012).

Dry weight (DW) of plant shoot organs (leaves, stems of the main tiller and the rest of the whole plant, and panicles) was measured after drying for 72 h in an oven at 70°C, during panicle development (PI + 3 weeks in GH-CNRS and GH IRRI; PI + 2 weeks in the field) and at heading (H; in GH-CNRS) or flowering (FLO; in the field). For each of this sampling main stem dry weight at H or FLO and MAT (MS DW H/FLO, MS DW MAT), shoot dry weight at FLO and MAT (Shoot DW H/FLO, Shoot DW MAT), main stem panicle dry weight at MAT (MS PDW MAT) and plant filled grain dry weight at MAT (Plant FGDW MAT) were measured.

At physiological maturity, the DW of all vegetative organs excluding root biomass was measured as well as MS PDW (after drying under the sun). The five plants harvested at maturity in GH were separated into panicles (after taken pictures for P-TRAP analysis), green leaf blades, senescent leaves, and productive stems (culms + sheaths). In the field, all the plants within a soil base area of 0.12 m^2^ per plot were harvested, that is three plants under LD and 12 plants under HD. They were then separated into panicles, green leaf blades, dead tissues, and productive stems. The panicles were hand-threshed and then the filled spikelets were separated from the unfilled by a densitometric column (in GH) or submerging the spikelets in the water (in the field).

PI was determined by dissecting and observing the main tiller of randomized collected plants (border plants for field experiment) from each unit treatment every second day when PI was close. The occurrence of PI was considered when the first row of floral primordial was visible on the shoot apex. Flowering (FLO) was determined within each unit treatment when an average of 75% spikelets per panicle of the main tiller exerted their anthers. Plants were considered at physiological maturity when 75% of the grains of the panicles had turned yellow and the texture was in dough stage.

#### Leaf area

In GH-CNRS, individual leaf area on the main stem was measured by using LI–3100C Area Meter (Lincoln, NE, USA). In the field and GH-IRRI, the length and maximum width of individual green leaf blades on the main stem was measured manually with a ruler, then leaf area was estimated as length × maximum width × 0.725 (Tivet et al., 2001). The measurement was done at PI + 3 weeks (four sampled plants in GH IRRI), and at FLO (four sampled plants in GH-CNRS and two sampled plants in the field) on the plants used for biomass measurement. The total area of all green leaves per plant was then measured by LI–3100C Area Meter in GH-CNRS and in the field.

#### Internode profile and anatomy

The length of each internode of the main tiller was measured at maturity in GH-CNRS and GH IRRI of sampled plants used for biomass measurement. In GH-CNRS, for anatomical observation purpose a middle part of 2 cm-long of peduncle and of internode-3 was sampled at FLO stage from the same plants used for biomass measurements. The samples were fixed in paraformaldehyde fixative solution and kept in desiccator overnight followed by dehydration with ethanol 70% for at least 24 h then conserved in the freezer prior to observation. The internodes were sliced into pieces of 60–80 μm with a Thermo Scientific Microm HM 650 V Vibration microtome. The pedunles were sliced into 75–90 μm after embedded in agarose 7% for 2 h with a Thermo Scientific Microm HM 650 V Vibration microtome.

In the field, internode profile was measured at FLO stage by sampling plants different from the plants used for biomass measurement. A 2 cm-long section of the peduncle and of the top internode was fixed in Formalin-Acetic-Alcohol (FAA) fixative solution until the date of sectioning. Prior to sectioning, the samples were dehydrate with ethanol 70% then sliced manually by razor blade.

The samples were then observed based on a high resolution imagery system (microscope Leica S8 APO equiped with camera QImaging MicroPublisher 3.3 in GH-CNRS and stereo microscope Olympus SZX7 equiped with camera Olympus DP71 in the field) to analyze the total and inner diameter as well as peripheric bundles number. In this study, peduncle is the uppermost internode between panicle neck node and node I, and top internode is the internode just below the peduncle, which is the same phytomer as flag leaf.

#### Carbon assimilation measurements

Actual assimilation rate (at homogenous level of light in the measurement chamber, i.e., 1500 or 1800 micromole m^−2^ s^−1^), was measured by using Portable Photosynthesis System (GFS–3000 WALZ) during panicle development (2–3 weeks after PI), the same time as biomass measurement for all experiments. The last ligulated leaf of the main tiller from three tagged plants (in GH-CNRS) and two tagged plants (in the field and GH-IRRI) in every replication was chosen for this measurement.

#### Non structural carbohydrates (NSC) analyses

NSC was analyzed during panicle development (2–3 weeks after PI) at the same time as C assimilation and biomass measurement in all experiments. In GH-CNRS, three plants per treatment (as three replications) were chosen homogenously for NSC analyses. In the field and GH-IRRI, two of sampled plants dedicated for biomass measurement were chosen for NSC analyses. In GH CNRS and GH IRRI trials, plants were dissected to sample the leaf blade of last ligulated leaf from the main stem. One base (only in GH CNRS) and one top internode (internode just below the peduncle) were sampled and immediately frozen in liquid nitrogen and store at −80°C until fine ground with a ball grinder (Mixer mill MM301, Retsch, Germany). In the field, all green leaves and internodes of the main tiller were sampled and dried for 72 h in an oven at 70°C before fine ground with a grinder (Thomas-Wiley Laboratory Mill Model 4, Thomas Scientific USA).

The method used for sugar content analysis in GH CNRS and GH IRRI was based on high performance liquid chromatography (HPLC). The sugars were extracted three times from 20 mg ground samples with 1 ml of 80% ethanol for 30 min at 75°C, and then centrifuged for 10 min at 10,000 rpm. Soluble sugars (sucrose, glucose and fructose) were contained in the supernatant and starch in the sediment. The supernatant was filtered in the presence of polyvinyl polypyrrolidone and activated carbon to eliminate pigments and polyphenols. After evaporation of solute with Speedvac (RC 1022 and RCT 90, Jouan SA, Saint Herblain, France), soluble sugars were quantified by high performance ionic chromatography (HPIC, standard Dionex) with pulsated amperometric detection (HPAE-PAD). The sediment was solubilized with 0.02 N sodas at 90°C for 1 h 30 min and then hydrolyzed with α-amyloglucosidase at 50°C, pH 4.2 for 1 h 30 min. Starch was quantified as described by Boehringer (1984) with 5 μL of hexokinase (glucose-6-phosphate dehydrogenase), followed by spectro-photometry of NADPH at 340 nm (spectrophotometer UV/VIS V-530, Jasco Corporation, Tokyo, Japan).

In the field IRRI, sugars were extracted two times from 200 mg ground samples with 7 ml of 80% ethanol for 10 min at 80°C, and then centrifuged for 5 min at 3000 rpm. The residue was washed with 5 ml of hot 80% ethanol three times and combined all washings with the supernatant. The residue was dried at 70°C for 24 h prior to starch assay. Total soluble sugars was determined through colorimetric by adding 5 ml anthrone to 0.5 ml aliquot (sugar extraction was diluted with 80% ethanol) then boiled for 10 min at 100°C. After vortex mix and cooling on ice bath for about 5 min, followed by spectro-photometry at 620 nm (DU 800 UV/Vis spectrophotometer, Beckman Coulter). Dried residue was dropped with absolute ethanol and added with 2 ml of acetate buffer then boiled for 3 h at 100°C. The tubes were cooled to 55°C and proceed to hydrolysis step by adding 1 ml acetate buffer and 1 ml amyloglucosidase, then vortex mix. After incubated for 24 h at 37°C, the hydrolysate (supernatant layer) was decanted and save combined with the residue that had been washed with 3 ml of distilled water. Starch was determined through colorimetric assay by adding enzyme Peroxidase Glucose Oxidase (PGO) to 0.6 ml aliquot (starch hydrolysis was diluted with distilled water), then followed by spectro-photometry at 450 nm (DU 800 UV/Vis spectrophotometer, Beckman Coulter) after incubated in the dark room for 30 min.

#### Physiological maturity and yield component

In GH IRRI four plants were harvested per replication and separated into panicles, green leaf blades, dead tissues and productive tillers then processed as two other experiments described in Adriani et al. (unpublished data). We determined yield components as panicle number per plant and plant FGDW.

#### Response rate

The rate of trait response to either *qTSN* introgression or to a reduced plant access to light was quantified as: (ref_value–mod_value)/ref_value; where ref_value is the trait value for the NIL (in the case of response to *qTSN* introgression) or for low plant access to light treatment (shading in GH-CNRS and HD in field-IRRI, in the case of response to low light quantification) and mod_value is the trait value for the parent or for full light treatment, i.e., control in GH-CNRS and LD in field-IRRI). Response rates are synthesized in **Table 3**.

#### Data analyses

The graphs describing plant morphogenesis, individual leaf area, grain production, relative NIL-P, and tillering rate relation to growth were represented with mean values and standard error (standard deviation divided by square root of the number of samples). Data of Tables 2, 3 and **Figures 3**, **4** were analyzed by an ANOVA procedure and mean comparisons between parent vs. NIL and between treatments for each pair of genotype were analyzed by Duncan's multiple range test using Microsoft® Excel 2010/XLSTAT-PRO statistical software (version 2014, Addinsoft, Inc., Brooklyn, NY, USA). SigmaPlot® Version 11.2 software (for Windows XP and below, copyright 2009–2010), Systat Software Inc. (Chicago, IL, USA) was used for plotting data and nonlinear regressions.

**Table 2:** **ANOVA of flag leaf area, tiller number at PI (Panicle Initiation), FLO (flowering; heading in GH-CNRS), MAT (grain physiological maturity), biomass related traits (DW, Dry Weight) at plant and main stem level at FLO and MAT for vegetative DW and at MAT only for panicle DW and FGDW (FG, Filled Grain)**.

**Source**	**Genetic background (G)**	**QTL**	**Treatment (T)**	**Replication**	**G × QTL**	**G × T**	**QTL × T**
**FLAG LEAF AREA**							
GH-CNRS	< 0.0001	0.0003	0.2902	0.9291	0.8243	0.5827	0.1783
Field	0.652	0.001	0.059	0.245	0.626	0.937	0.212
**TILLER NUMBER AT PI**							
GH-CNRS	0.026	0.055	0.266	0.235	0.721	0.922	0.627
Field	0.097	0.0005	< 0.0001	0.066	0.455	0.084	0.054
**TILLER NUMBER AT FLO**							
GH-CNRS	0.937	0.187	0.812	0.973	0.479	0.812	0.581
Field	0.099	0.079	< 0.0001	0.489	0.980	0.056	0.367
**TILLER NUMBER AT MAT**							
GH-CNRS	0.959	0.720	0.574	0.924	0.878	0.878	0.959
Field	0.001	0.021	< 0.0001	0.038	0.186	0.008	0.584
**MS DW FLO**							
GH-CNRS	0.005	0.003	< 0.0001	0.154	0.224	0.035	0.502
Field	0.002	< 0.0001	< 0.0001	0.257	0.192	0.870	0.022
**MS DW MAT**							
GH-CNRS	0.0002	0.002	0.327	0.807	0.014	0.002	0.303
Field	0.126	0.165	0.003	0.949	0.657	0.452	0.475
**SHOOT DW FLO**							
GH-CNRS	0.492	0.224	< 0.0001	0.332	0.197	0.821	0.444
Field	0.0002	0.126	< 0.0001	0.868	0.729	0.013	0.112
**SHOOT DW MAT**							
GH-CNRS	0.080	0.180	0.122	0.565	0.478	0.095	0.502
Field	0.002	0.096	< 0.0001	0.113	0.302	0.010	0.769
**MS PDW MAT**							
GH-CNRS	0.772	0.0001	< 0.0001	0.619	0.249	0.678	0.209
Field	0.041	0.004	0.001	0.410	0.258	0.166	0.539
**PLANT FGDW MAT**							
GH-CNRS	0.089	< 0.0001	< 0.0001	0.946	0.073	0.863	0.922
Field	0.048	0.083	< 0.0001	0.140	0.155	0.078	0.443

**Table 3:** **Response rate of traits to QTLs introgression in each pair of isoline in a given treatment (in the field-IRRI, LD is for Low Density, HD is for high density) and genetic background (IR64 and IRRI146) (A), to access to light in each trial (field-IRRI, GH-CNRS) (B), for each genotype (parent, NIL, NIL1)**.

**A**										
**Traits**	**IR64-QTL effect**		**IRRI146-QTL effect**		**IR64-QTL effect (NIL)**		**IR64-QTL effect (NIL1)**		**IRRI146-QTL effect**	
	**Control**	**Shading**	**Control**	**Shading**	**LD**	**HD**	**LD**	**HD**	**LD**	**HD**
Flag leaf area	0.530	0.181	1.091	0.478	0.265	0.156	0.339	0.046	0.291	0.231
Tiller number at PI	−0.167	−0.085	−0.259	−0.160	−0.196	−0.183	−0.263	−0.327	−0.295	−0.161
Tiller number at FLO	−0.014	0.076	−0.147	−0.137	−0.124	−0.069	−0.292	−0.240	−0.072	−0.192
Tiller number at MAT	0.000	0.026	0.041	0.030	−0.052	−0.213	−0.069	−0.244	−0.223	−0.190
MS DW FLO	0.168	0.255	0.126	0.060	0.410	0.579	0.608	0.454	0.358	0.118
MS DW MAT	0.177	0.600	0.082	0.030	0.204	0.018	0.121	0.051	0.290	0.121
Shoot DW FLO	0.030	0.281	0.010	−0.020	0.037	0.068	0.128	0.103	0.133	−0.147
Shoot DW MAT	0.068	0.155	−0.004	0.080	−0.023	−0.179	0.025	−0.040	−0.141	−0.143
MS PDW MAT	0.346	1.158	0.275	0.326	0.058	−0.022	0.271	0.507	0.386	0.129
Plant FGDW MAT	0.310	0.948	0.195	0.198	−0.022	−0.097	0.131	0.075	−0.210	−0.232
**B**										
**Traits**	**IR64-light effect**		**IRRI146-light effect**		**IR64-density effect**			**IRRI146-density effect**		
	**Parent**	**NIL**	**Parent**	**NIL**	**Parent**	**NIL**	**NIL1**	**Parent**	**NIL**	
Flag leaf area	0.300	0.004	0.328	−0.061	0.024	−0.064	−0.200	−0.057	−0.101	
Tiller number at PI					−0.635	−0.629	−0.667	−0.717	−0.664	
Tiller number at FLO	−0.096	−0.014	0.176	0.190	−0.659	−0.637	−0.634	−0.540	−0.600	
Tiller number at MAT	0.013	0.040	0.050	0.039	−0.588	−0.656	−0.665	−0.694	−0.681	
MS DW FLO	−0.429	−0.386	−0.300	−0.338	−0.281	−0.195	−0.349	−0.157	−0.305	
MS DW MAT	−0.326	−0.084	0.190	0.138	−0.091	−0.232	−0.148	−0.210	−0.313	
Shoot DW FLO	−0.331	−0.168	−0.230	−0.250	−0.708	−0.699	−0.714	−0.674	−0.754	
Shoot DW MAT	−0.184	−0.118	−0.030	0.046	−0.678	−0.729	−0.698	−0.735	−0.735	
MS PDW MAT	−0.607	−0.369	−0.499	−0.479	−0.319	−0.370	−0.192	−0.048	−0.224	
Plant FGDW MAT	−0.589	−0.388	−0.454	−0.453	−0.733	−0.754	−0.746	−0.772	−0.778	

*Gray columns indicate no treatment effect as shading in GH-CNRS just imposed at PI*.

## Results

### QTL effects on plant morphogenesis

#### Under full light conditions

**Figure 1:**
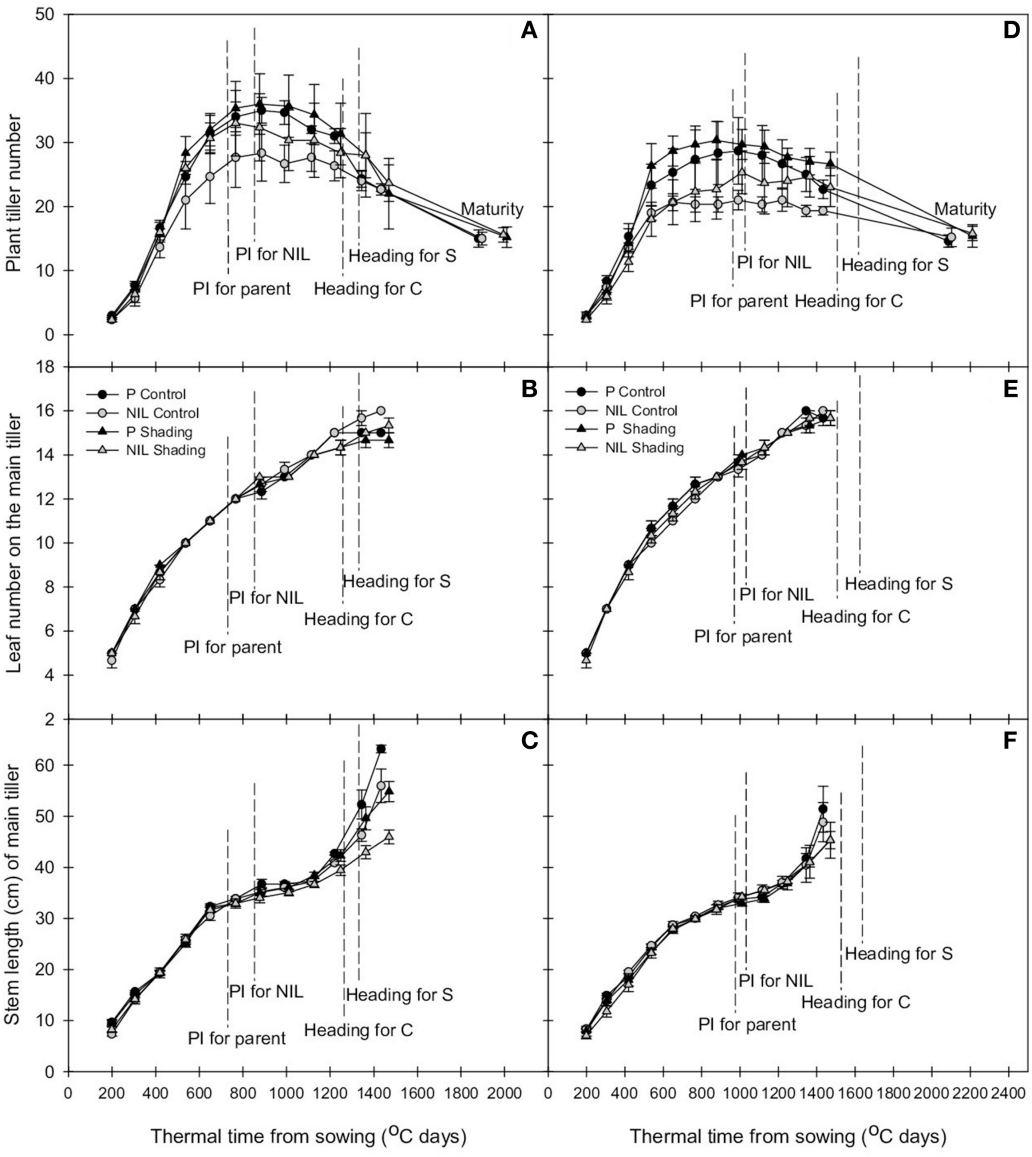
**Change with thermal time of plant tiller number (A,D), leaf number on the main tiller (B,E), and stem length of the main tiller (C,E) of parent (black) and NIL (gray) in IR64 (A–C) and IRRI146 (D–F) background, under control (C) and shading (S) in GH-CNRS trial**. The values are mean ± SE. *n* = 3.

**Figure 2:**
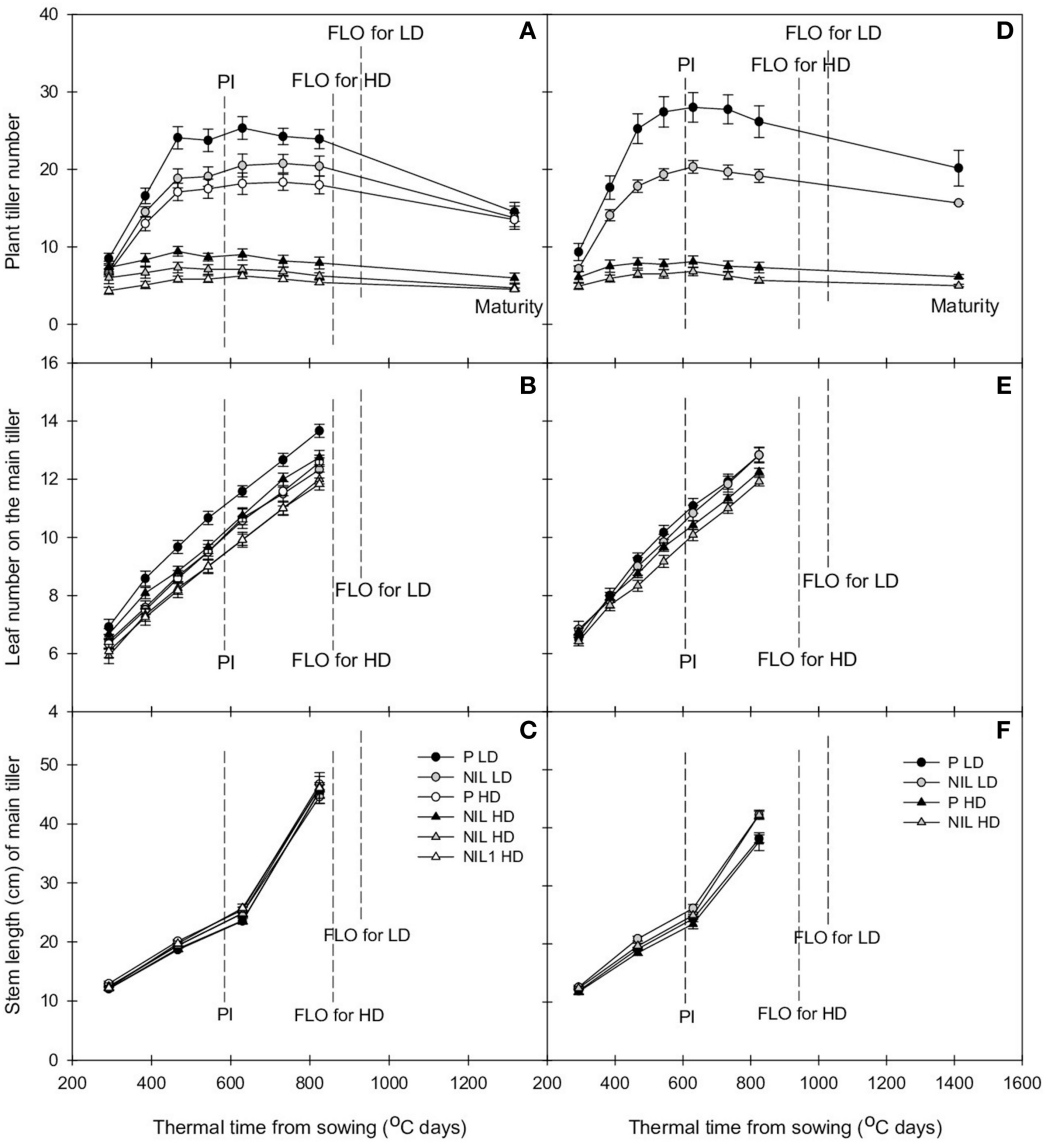
**Change with thermal time of plant tiller number (A,D), leaf number on the main tiller (B,E), and stem length of the main tiller (C,E) of parent (black) and NIL (gray) in IR64 (A–C) and IRRI146 (D–F) background, under low density (LD) and high density (HD) in field trial**. The values are mean ± SE. *n* = 4.

Tiller dynamic is delayed in the presence of *qTSN*. This effect was observed at early tillering stage, i.e., starting at 400°C days (accumulation of thermal time from sowing) when eight leaves had appeared on the main stem. This was true for both genetic backgrounds and experiments (Figures 1A,D, 2A,D; Figures S1B, S2B) and resulted in a reduced tiller number at PI that was however only significant in the field (*P* < 0.001, see Table 2 for ANOVA). In IR64 background, this reduction rate ranged from 8.5 to 16.7% in GH-CNRS and the highest reduction rate was observed for NIL1 in the field (between 26.3 and 32.7%; Table 3A). In IRRI146 background, the reduction rate ranged from 16 to 25.9% (GH CNRS) and up to 29.5% in the field (Table 3A). This reduction was associated with a smaller rate of tiller abortion until MAT for the NILs compared to the parents, resulting in a progressive convergence of tiller number of parents and NILs at MAT (Figures 1A,D, 2A,D; Figure S1B; Tables 2, 3). Nevertheless, fertile tiller number in IRRI146 background in the field kept smaller at MAT in the NIL compared to the parent (22.3 and 19% of reduction under LD and HD, respectively) as well as for the IR64 parent in HD treatment (21.3 and 24.4% less tillers in NIL and NIL1, respectively, compared to the parent).

The effect of *qTSN* on leaf appearance rate and final leaf number on the main stem was in general weak and not homogenous across treatments. The *qTSN4* had a depressive effect on final leaf number on the main tiller in the field, although less pronounced in IRRI146 background (Figures 2B,E; Figure S2A). This could be related to a slightly lower rate of leaf appearance considering the similar duration of the vegetative phase. In contrast, in GH-CNRS, higher leaf number (one more in average) in the presence of *qTSN4* was observed in IR64 background only, which was appreciable at the end of panicle development (about 1300°C days, at time of appearance of leaf 14; Figure 1B). This can be related to the fact that the vegetative phase was slightly longer in the NIL (later PI), of approximately one phyllochron, i.e., duration between the appearance of two consecutive leaves (Figures 1B,E; Figure S1A).

Similarly to that observed with leaf number, *qTSN* effect on stem length (considered here as the successive internodes excluding the peduncle) also differed from GH-CNRS to field-IRRI trials as well as between genetic backgrounds. An appreciable decrease in final stem length was observed in IR64 background in the presence of *qTSN* in GH-CNRS (Figure 1C), whereas in the field no *qTSN* effect on stem length was observed (Figure 2C; Figure S2C). No clear difference was observed in IRRI146 background (Figures 1F, 2F; Figure S1C). The effect of *qTSN* on peduncle length differed with respect to the genetic background and the experiment. In GH-CNRS, the peduncle (the internode bearing the panicle) was significantly longer in the presence of *qTSN4* in IR64 background (not presented) but it was the opposite in IRRI146 background (Figure S1E), which was confirmed in GH-IRRI (data not presented). In the field, the peduncle was shorter in the presence of *qTSN12* in IR64 background (Figure S2E), whereas no effect was observed in IRRI146 background (not presented). In IRRI146 background in GH-CNRS, no significant difference in stem length was observed between parent and NIL until heading (Figure S1C), but thereafter *qTSN4* positively affected the length of the top three internodes located just below the peduncle. The peduncle was, however, shorter in the NIL compared to the parent (Figure S1E).

More stable effect of *qTSN* could be observed on peduncle anatomy and thickness for both genetic backgrounds and trials. The increase of peduncle thickness was 44% in IRRI146 background in GH-CNRS (Figure S1F), and 14% in IR64 background (not presented). In the field, the increase of peduncle thickness in the presence of *qTSN* was 20% in IR64 background (Figure S2F) and 17% in IRRI146 background (not presented). The characteristics of the peduncle were associated with thicker top internode (in the third internode below the peduncle in GH-CNRS, Figure S1F; in top internode in the field, Figure S2F) and higher number of vascular bundles in the peduncle (data not presented) in the NILs compared to the parents.

**Figure 3:**
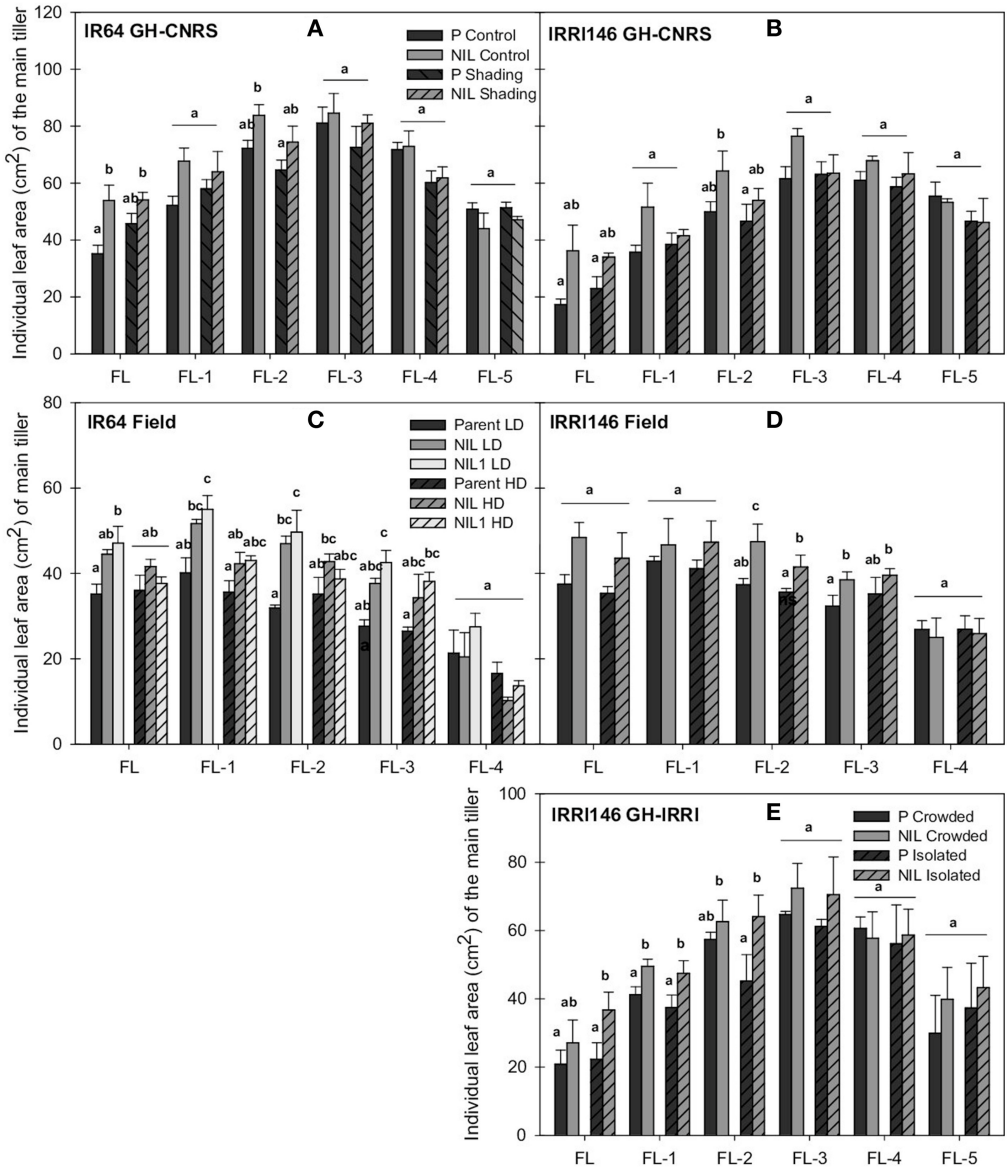
**Individual leaf area of main tiller (FL, flag leaf; FL-1–FL-5, one to five leaves below the flag leaf) at flowering (FLO) of parent (black), NIL (gray), and NIL1 (white) in IR64 background (A) and IRRI146 background (B) in GH-CNRS under control and shading, in IR64 background (C) and IRRI146 background (D) in the field under low density (LD) and high density (HD), in IRRI146 background (E) in GH-IRRI under crowded and isolated plants**. The values are mean ± SE. Results of Duncan test for multiple comparisons of each genotype per treatment at 5% level are shown in the letters above the bars. *n* = 4 in GH-CNRS and filed-IRRI, *n* = 3 in GH-IRRI.

A positive effect of *qTSN* on leaf area was observed for the flag leaf (FL) (significant in IR64 background for both trials) and the two to three leaves below the flag leaf (FL-3 or FL-2), but it was more pronounced for FL (Figure 3). In GH-CNRS, the increase was 53% for IR64 background (Figure 3A; Table 3A) and 109% in IRRI146 background (Figure 3B; Figure S1D; Table 3A), which was mainly explained by an increase of leaf length, whereas the width was not affected (data not presented). In the field, *qTSN* effect on the leaf area of the top leaves was already expressed from FL-3 upward for both genetic backgrounds (and significant for all these leaves only in NIL1) (Figures 3C,D), with 34% of increase in IR64 background (Figure S2D; Table 3A for QTL effect in NIL1) and 29% of increase in IRRI146 (Table 3A). In GH IRRI it was expressed and significant from FL-2 upward (Figure 3E). In the field and GH IRRI, the positive effect of *qTSN* on individual leaf size was mainly supported by the width (data not presented) rather than the length.

#### Under low light conditions

Traits related to plant growth and development were more affected by the treatment in the field. This can be easily explained by the fact that treatments were established by planting density from sowing onwards in the field, while shading treatment in GH-CNRS was imposed only from PI time to heading. Accordingly, early tillering (Figures 1A,D, 2A,D; Tables 2, 3B for the rate of trait plasticity in response to light treatment) and leaf appearance rates (Figures 1B,E, 2B,E) were poorly affected by shading in GH-CNRS. By contrast stem length was more affected by shading in GH-CNRS, i.e., decreased, as observed in field-IRRI but only in HD treatment in IRRI146 isolines.

Peduncle and internode thickness were also reduced under low access to light in both experiments but only in the NILs, whereas they were not modified in the parents (not presented). Individual leaf size was almost not affected by the reduction of incoming light. In GH-CNRS, main tiller FL size of that parents of both backgrounds increased by about 30% under shading, whereas under the same conditions it was maintained or reduced with the NILs. In the field, slight increase in FL area due to high density was observed only for the parent of IR64 background (2%) (Figures 3A,D; Table 3B).

As observed under full light conditions, tiller number was reduced with *qTSN* under low plant access to light (shading in GH-CNRS, HD in the field), however, the difference between NIL and parents was not as strong as observed under higher plant access to light (Figures 1A,D, 2A,D; Table 3B), and final tiller number was similar between NILs and parents at maturity. The *qTSN* effect on leaf appearance (positive for IR64 background in GH-CNRS; negative in the field; unchanged for IRRI146 background in both trials) and stem elongation (negative or unchanged) was similar than that observed in full light conditions (Figures 1, 2). The *qTSN* positive effect on individual leaf size under low plant access to light was appreciable but less pronounced than that observed under high access to light, as the low access to light increased top leaf size of the parents but not of the NILs (Figure 3; Table 3B). No QTL effect was observed on the traits related to peduncle and internode anatomy under low light condition (not presented).

#### Biomass, leaf area and grain productions

**Figure 4:**
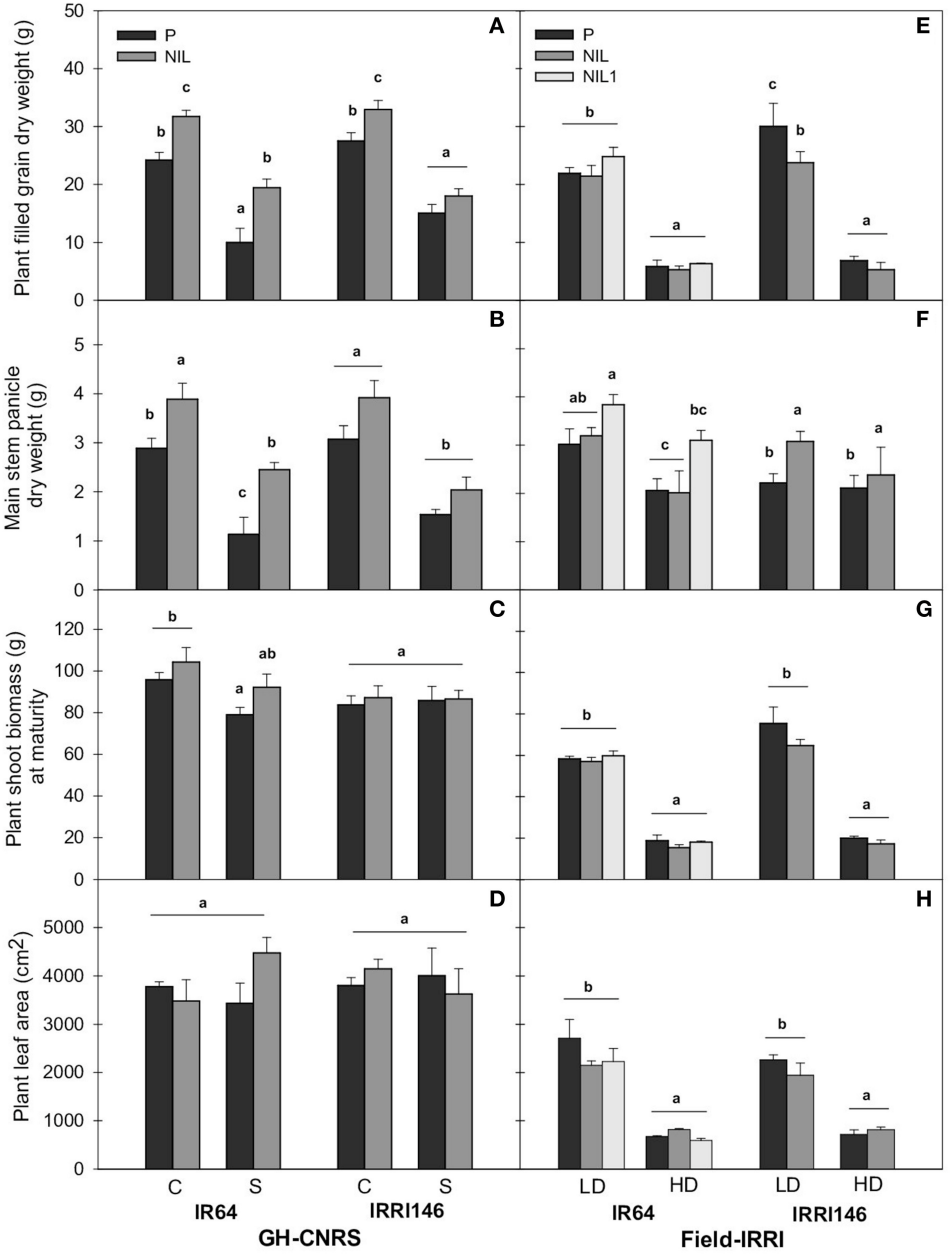
**Plant filled grain dry weight (A,E), main stem panicle dry weight at maturity (B,F), plant shoot biomass at maturity (C,G), plant green leaf area at flowering (D,H) under control (C), and shading (S) in GH-CNRS (A–D), under low density (LD), and high density (HD) in field-IRRI (E–J)**. The values are mean ± SE. Results of Duncan test for multiple comparisons of each genotype per treatment at 5% level are shown by the letters above error bars. *n* = 5 at maturity and *n* = 4 at flowering in GH-CNRS, *n* = 4 in field-IRRI.

At plant level, in GH-CNRS, *qTSN4* increased plant final grain production (FGDW) which was significant in all cases (Table 2) except for IRRI146 background under shading (Figure 4B; Table 3A). Meanwhile, plant shoot biomass was not affected by *qTSN*4 neither at PI and FLO (heading in GH-CNRS) (not presented) nor at physiological maturity (Figure 4C; Tables 2, 3A). In the field, *qTSN* poorly affected grain and straw biomass and not systematically in a positive way. An increase could be observed only in IR64 background in plant grain production but this was not significant. The *qTSN* effect was even significantly negative on plant grain production in IRRI146 under LD (Figure 4E; Table 3A). These contradictory results between GH-CNRS and field conditions regarding *qTSN4* effect on grain production at plant level were also observed in GH-IRRI where no significant QTL effect on plant grain production was observed (results not presented). The leaf area per plant at flowering time was unaffected by *qTSN* but the distribution of leaf area was modified in a way that individual leaves were larger but fewer in the presence of *qTSN*. This was true for both genetic backgrounds in both trials (Figures 4D,H). However, plant leaf area was affected by light conditions in the field, where it was higher in full light (LD) compared to low light (HD) condition.

At main stem level, panicle dry weight at MAT was increased by *qTSN* in both genetic backgrounds and trials (Figures 4B,F; Table 2; *P* < 0.01). This increase was more pronounced and systematic in GH-CNRS. It was generally higher under low light conditions for IR64 background (115% under shading and 51% under HD in NIL1, but no effect observed in the NIL of IR64 in field-IRRI, Table 3A), whereas it was more homogenous among treatments for IRRI146 (Table 3A). The main stem panicle DW was systematically reduced by low access to light and no *qTSN* × treatment interactions were observed (Tables 2, 3B). The main stem DW at FLO was systematically increased by *qTSN* (*P* < 0.01, Table 2), and this was generally stronger in IR64 background compared to IRRI146 and more particularly in field-IRRI. This *qTSN* effect was not maintained until MAT as no significant difference for main stem DW between parents and NIL could be observed at that stage (Tables 2, 3). Low plant access to light systematically reduced main stem DW at FLO, more particularly in GH-CNRS (Table 3). This was maintained until maturity only in the field-IRRI as no more significant treatment effect was observed in the GH-CNRS at this time (Tables 2, 3). No *qTSN* × treatment interaction for main stem DW was observed, neither at MAT or FLO, except in field-IRRI at FLO (Table 2).

#### Relationship between tillering dynamics and main stem growth rate

**Figure 5:**
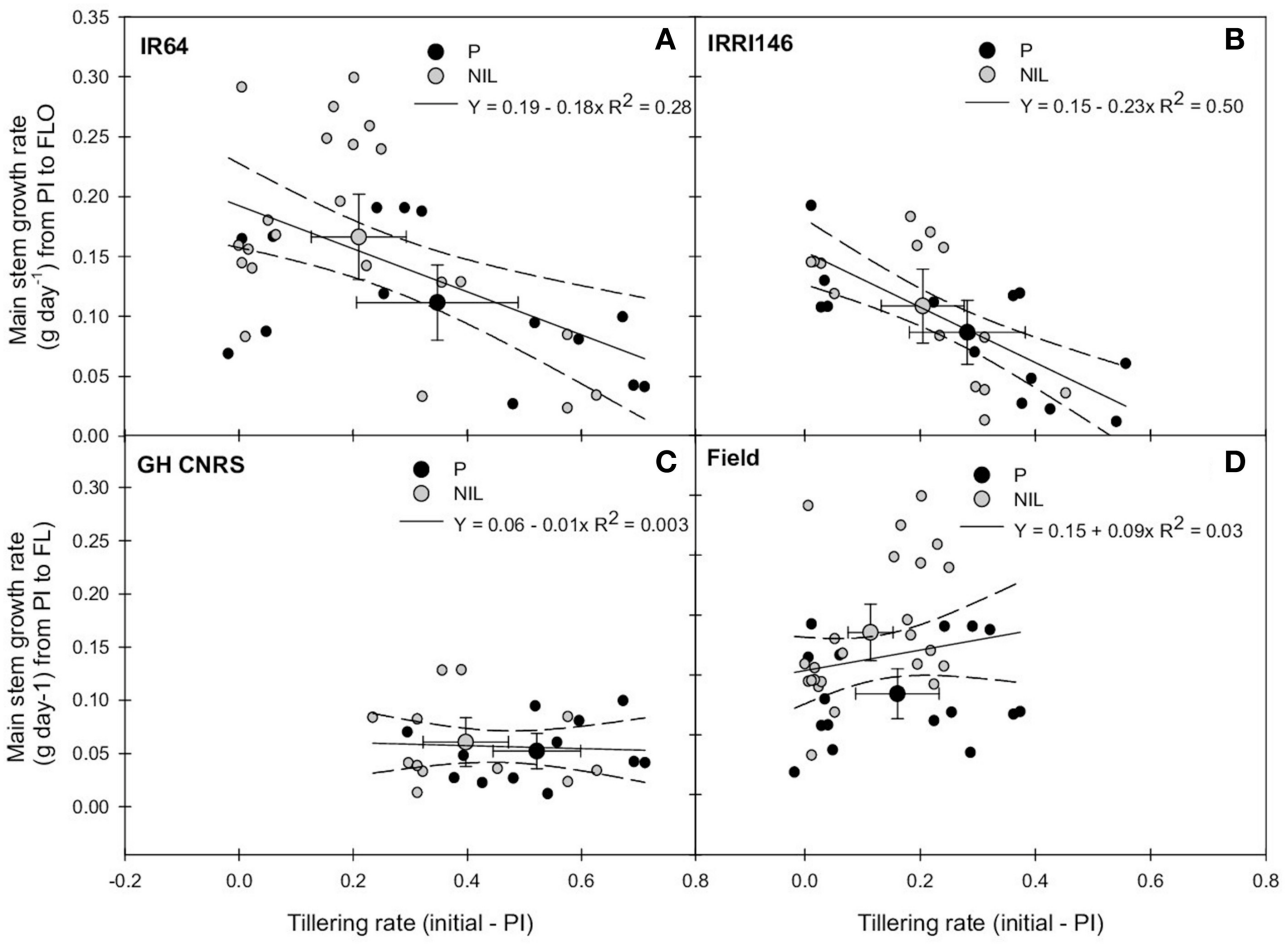
**Relationship between tillering rate from initial measurement to panicle initiation (PI) and main stem growth rate from PI to flowering (FLO) of the parent (black symbol) and the NIL (gray symbol), in IR64 background in GH-CNRS and field trials (A) in IRRI146 background in GH-CNRS and field trials (B) in GH-CNRS in IR64 and IRRI146 backgrounds (C) in field-IRRI experiment in IR64 and IRRI146 backgrounds (D)**. The values are mean ± SE. Regression curves are associated with confidence interval at *P* = 0.05. *n* = 40 for IR64 background and field-IRRI trial, *n* = 32 for IRRI146 background and GH-CNRS trial for regression curve.

Overall, above-mentioned results pointed out two key nodes of regulation of plant phenotypes due to *qTSN* introgression, namely: (i) tillering and tiller number, generally reduced by *qTSN* and (ii) main stem biomass (either before or after FLO), generally increased by *qTSN* (Table 3A). In association with the opposite effect of *qTSN4* on these two traits, no additional difference was observed between parents and NILs regarding the resulting plant shoot DW at FLO and MAT. Results of plant FGDW were, however, dependent on cropping conditions (Figures 4A,D; Table 3A). In order to further explore the relationship between tillering and main stem DW, the change of main stem growth rate during panicle development (PI–FLO period) was plotted against tillering rate before PI. A negative correlation could be observed between these variables, stronger in IRRI146 (*R*^2^ = 0.5) than in IR64 (*R*^2^ = 0.28) backgrounds (Figures 5A,B). Interestingly, the average value for the NIL was systematically at a higher position on the y-axis (with reference to main stem growth rate from PI to FLO) compared to that of the parents. However, the correlation disappeared when analyzing the same relationship in each experiment separately (Figures 5C,D).

**Figure 6:**
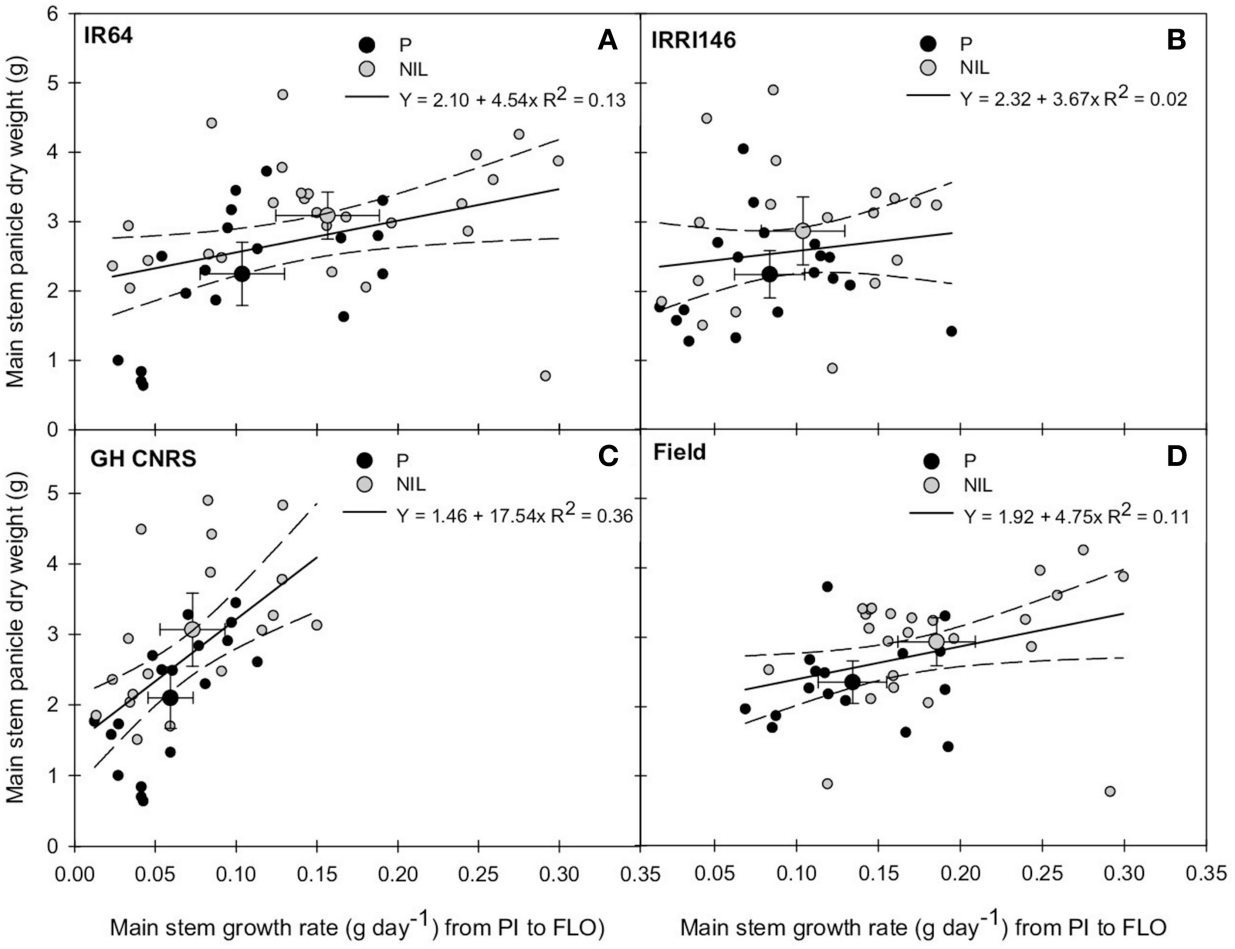
**Relationship between main stem growth rate from panicle initiation (PI) to flowering (FLO) and main stem panicle dry weight at maturity of the parent (black symbol) and the NIL (gray symbol), in IR64 background in GH-CNRS and field trials (A) in IRRI146 background in GH-CNRS and field trials (B) in GH-CNRS in IR64 and IRRI146 backgrounds (C) in field-IRRI experiment in IR64 and IRRI146 backgrounds (D)**. The values are mean ± SE. Regression curves are associated with confidence interval at *P* = 0.05. *n* = 42 for IR64 background and field-IRRI trial, *n* = 34 for IRRI146 background and GH-CNRS trial for regression curve.

In order to evaluate whether this early trade-off between tillering and main stem growth rate (from PI to FLO) could impact grain production, main stem growth rate from PI to FLO was plotted against main stem panicle DW at maturity. This is presented in Figures 6A,B, showing a slightly positive correlation between these variables (*R*^2^ = 0.12) when analyzing data from parents and NILs together, whereas there was no correlation in IRRI146 (*R*^2^ = 0.02) background. This positive correlation was getting even stronger when considering trials separately (Figures 6C,D), in particular at GH-CNRS. In all situations, the average of NIL values showed a higher main stem growth rate from PI to FLO related to a bigger panicle DW on the main stem at maturity.

**Figure 7:**
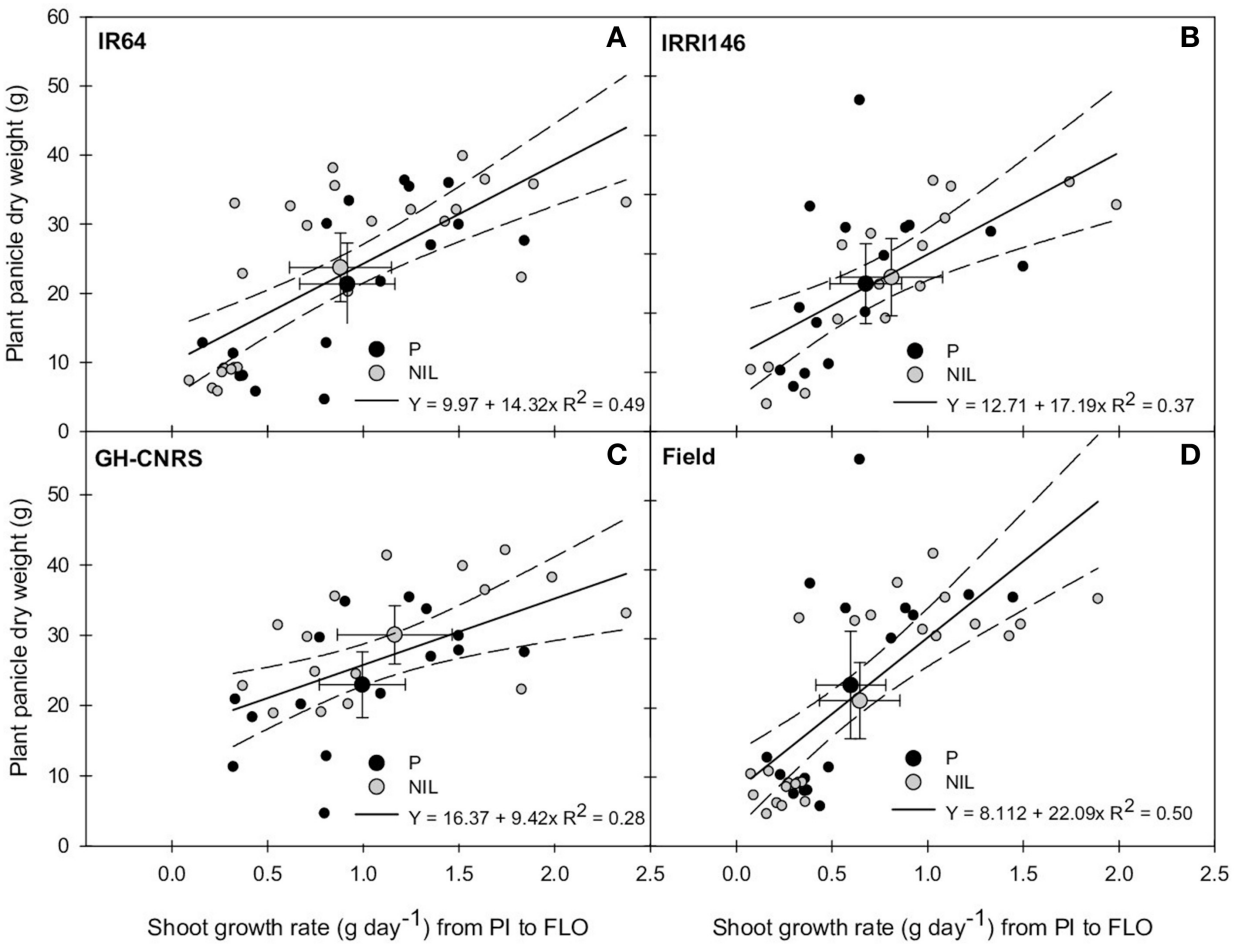
**Relationship between plant shoot growth rate from panicle initiation (PI) to flowering (FLO) and plant panicle dry weight at maturity of the parent (black symbol) and the NIL (gray symbol), in IR64 background in GH-CNRS and field trials (A) in IRRI146 background in GH-CNRS and field trials (B) in GH-CNRS in IR64 and IRRI146 backgrounds (C) in field-IRRI experiment in IR64 and IRRI146 backgrounds (D)**. The values are mean ± SE. Regression curves are associated with confidence interval at *P* = 0.05. *n* = 42 for IR64 background and field-IRRI trial, *n* = 34 for IRRI146 background and GH-CNRS trial for regression curve.

The relationship between main stem and plant shoot growth rates from PI to FLO was thereafter explored, and no significant correlation was observed (not presented). Meanwhile, plant grain production was positively correlated to the whole plant shoot growth rate from PI to FLO (Figure 7). This correlation was higher in IR64 (*R*^2^ = 0.49) than in IRRI146 (*R*^2^ = 0.37) background and in field-IRRI (*R*^2^ = 0.50) than in GH-CNRS (*R*^2^ = 0.28) trial. Interestingly, with respect to this correlation, the NILs performed better than parents only in GH-CNRS whatever the genetic background (Figure 7C), i.e., in the cropping situation where the trade-off between tillering and main stem growth rates was the lowest for the NIL and the nearest from that of parents (Figure 5C). It can be mentioned that plant grain production was not correlated to main stem growth rate from PI to FLO (Figure S3).

#### Carbon assimilation and sugar related traits

**Figure 8:**
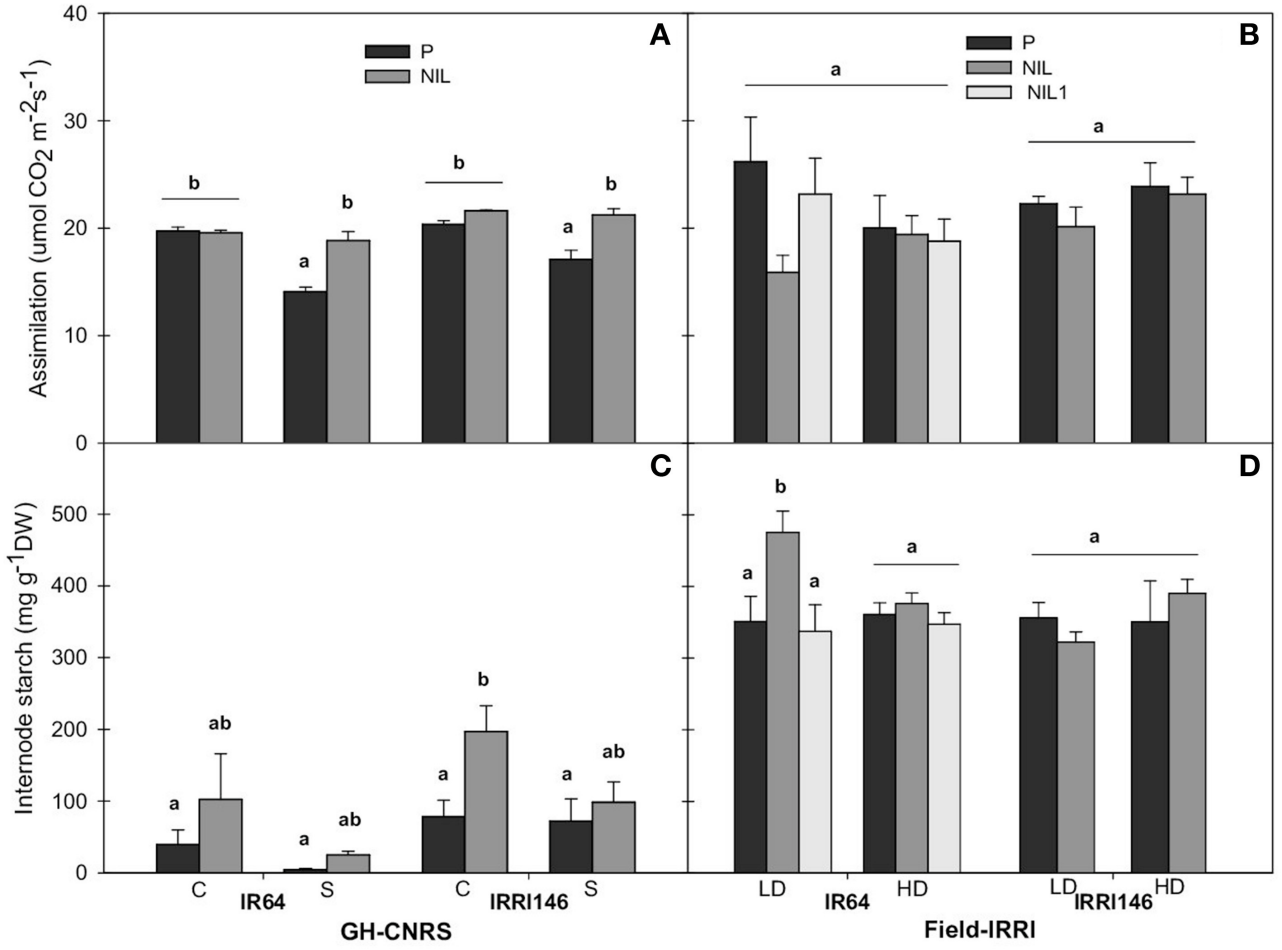
**Carbon assimilation (A,B) and internode starch concentration during panicle development (C,D) of parent (black), NIL (gray) and NIL1 (white) under control (C) and shading (S) in GH-CNRS (A,C), under low density (LD) and high density (HD) in field-IRRI (B,D)**. The values are mean ± SE. Results of Duncan test for multiple comparisons of each genotype per treatment at 5% level are shown in the letters above the bars. *n* = 3 in GH-CNRS, *n* = 4 in field-IRRI.

In order to identify whether the difference in main stem growth rate was associated with a particular metabolic pattern, starch and net assimilation rate at ambient CO_2_ concentration of 400 ppm were quantified during panicle development. In GH-CNRS, *qTSN4* enhanced assimilation only in shading treatment by 33% for IR64 and 24% for IRRI146 background (Figure 8). Shading significantly reduced assimilation in parent lines, 29 and 16% for IR64 and IRRI146 background, respectively, whereas in the NILs, assimilation was maintained under shading (Figure 8A). In the field, no significant *qTSN* and treatment effect was observed on assimilation, even if it was slightly decreased with *qTSN* in both backgrounds (Figure 8B). In GH-IRRI, *qTSN4* increased assimilation under low light (crowded plants) conditions but it was the opposite under full light (isolated plants) conditions (Figure S4A).

Internode starch content increased in the presence of *qTSN* for both backgrounds across the trials (Figures 8C,D; Figure S4B). The *qTSN4* effect was stronger under full light conditions (control in GH-CNRS, LD in the field, isolated in GH-IRRI), and significant in IRRI146 background in GH-CNRS (18%) (Figure 8C) and IR64 background in the field (11%) (Figure 8D). Similar trend was observed in leaf blade, with no significant effect of *qTSN* across the trials (not presented). However, in most cases, leaf starch was strongly reduced under low plant access to light.

## Discussion

The isolines (NIL) used in this study carried *qTSN4* or *qTSN12*, known to enhance leaf and panicle sizes but to reduce panicle number in some environmental situations (Fujita et al., 2013; Okami et al., 2015). The present study aimed at better characterizing this trade-off by comparing the NILs to their recurrent parents IRRI146 and IR64 regarding morphogenesis and C source-sink balance along the whole plant cycle and their behavior under low access to light.

### The QTSN affects rice morphogenesis and physiology at earlier stage than expected

A reduction of the rate of tiller emergence before PI (as early as 400°C days) was observed in this study in the presence of *qTSN* for both genetic backgrounds under both treatments. This was, however, not addressed in previous studies (Fujita et al., 2012, 2013) where the breeders mainly focused their attention on latter traits measured between flowering and maturity. Nevertheless, Okami et al. (2015) confirmed that tiller number was reduced with *qTSN4* under drought stress at vegetative stage as tillering rate per unit of above-ground biomass of the NIL was lower than that of the parent, which is in line with the present study. But in contrast to the present study, no difference between P and NIL was observed regarding tillering dynamics under well water conditions (Okami et al., 2015) and the ratio of main stem leaf area to tiller number (Okami et al., 2012). Interestingly, in the present study, main stem growth rate between PI and FLO was inversely proportional to tillering rate before PI (Figure 5). Considering that reduction in tillering rate is expected to provide more assimilate available to the growing stems, the difference in main stem growth rate appears as a consequence of the change in early tillering rate. Several hypotheses subtending this correlation can be raised. On one hand, it can be hypothesized that *qTSN* implies a higher apical dominance due to hormonal signals, e.g., in relation to strigolactone (Jamil et al., 2012) or other hormones (ABA, IAA, GA3; Liu et al., 2011). This may be associated with a higher sensitivity to the red/far red ratio within the canopy maybe brought by *qTSN*. Indeed cessation of tillering in crops has been widely reported to be correlated with the increase, with crop age, in red/far-red ratio sensed by the plant within the canopy, even before any C limitation occurred within the plant (Ballaré and Casal, 2000; Ugarte et al., 2010). On the other hand, sink strength of growing stems should be higher at early stage in the presence of QTL, due to the initiation and pre-dimensioning of larger organs at meristem level, which may decrease tiller bud outgrowth. In this latter case, tillering would be reduced by a competition (or at least a signaling of competition) for resources active meristem and organs and tiller bud outgrowth, as pointed out by Rebolledo et al. (2012) across japonica rice genetic diversity. Whatever the hypothesis, the reduction in early tillering rate coincides with the appearance of leaf 8 in GH (rather leaf 7 in the field) so with the initiation of leaf 10 or 11, based on the developmental pattern established by Nemoto et al. (1995). Interestingly in GH, the leaves with larger area in the presence of QTL were the 3–4 top leaves of the main stem, so those initiated right after leaf 11.

The size of organs was enhanced by *qTSN*4 on the main stem, from 3 to 4 leaves below the flag leaf up to the panicle. In addition to individual leaf area from the upper phytomers, internode length, and internode and peduncle thickness, were also enhanced with *qTSN*, while peduncle length and plant height were reduced. This finding was also reported in a recent study using the same genetic materials but comparing genotype behavior under drought and well-watered conditions (Okami et al., 2015). This is in line with Fujita et al. (2013) using the same genetic materials and Wu et al. (2011) using other genetic materials revealing that rice plants with larger culm diameter exhibited longer and wider flag leaf, more grains per panicle, as well as lower tiller number. Furthermore, Liu et al. (2008) confirmed that a thicker peduncle plays an important role in the determination of panicle size and grain yield potential.

This behavior in the presence of *qTSN* may be thus related to a stronger expression of apical dominance as the changes in organ size could not be detected at plant level: plant leaf area and shoot biomass at flowering were not different between parents and NILs, mainly due to the compensation between organ size and number. Nevertheless, the increase in C assimilation (in GH under shading) and in sugar (in particular starch) storage in stems (only in GH) suggested that *qTSN* enhances C availability within the main tiller at least during panicle development. Whether this is related to leaf anatomy and the elaboration of plant leaf area based on larger, thicker but fewer leaves needs to be confirmed. The fact that *qTSN4* co-localizes with NAL1, a gene involved in leaf anatomy, veining pattern and carboxylation, provides further insight to this *QTL* (Qi et al., 2008).

### The QTSN early trade-off between tiller and main stem growth partially explains its environment dependent effect on plant grain production

The reduction of early tillering and its benefits for main stem growth rate in the presence of *qTSN* was dependent on the genetic background and the environment: it was more pronounced in GH-CNRS and in IR64. In addition, the positive effect of *qTSN* on plant grain production was mostly revealed in GH-CNRS while it was weak or inexistent in the field. This can be explained by the fact that in GH-CNRS fertile tiller (i.e., panicle) number was less reduced by *qTSN4* compared to that observed in the field, while its effect on panicle size was strong. The low effect in the field cannot be totally interpreted, but it is in line with a previous study (Okami et al., 2014) that revealed the absence of difference in grain yield between parents and NILs of IR64 background during three summer seasons under flooded and aerobic conditions in Japan. The same authors, however, reported some differences under aerobic conditions in one season with low nitrogen supply (90 kg N ha^−1^). These results support the present study suggesting that a positive effect of the *qTSN* may be expressed depending on the cropping environment, where stressing environments (low light, low N, low soil exploration as in pots in greenhouse) should favor the benefits of *qTSN* on panicle size and, if ever, on grain production.

### Earlier tiller cessation and its implication for yield potential

Increasing yield potential by inducing an earlier cessation of tiller production was already proposed in previous studies comparing performances of hybrids and inbreds (Bueno and Lafarge, 2009; Lafarge et al., 2010). An earlier cessation of tillering was then related to an earlier biomass accumulation within reproductive stems and to a higher biomass remobilization from internodes to panicles, as also promoted by a higher sensitivity to the red/far red ratio within the canopy (Ballaré and Casal, 2000). Interestingly, the present study also highlighted the correlation between an earlier tillering cessation and higher main stem biomass growth, in association with a larger sink size. In the present study, however, these correlations were weak at plant level in the field particularly, highlighting the complexity of the GxE interactions and trade-offs underlying the *qTSN* effect on plant grain production. It will be interesting to pay more attention on other fertile tillers to better understand *qTSN* impact on the whole plant growth and grain production. Nevertheless, this study reinforces the interest of developing genotypes optimizing tillering dynamics as long as yield potential is concerned.

## Conclusion

The *qTSN* was confirmed in this study as a QTL potentially increasing panicle size due to an increase in stem growth rate, and in the size of the top leaves and internodes, at least at the main stem level. However, the trade-off between panicle size and panicle number was identified as the key node modulating the environment-dependent *qTSN* positive effect on plant grain production. This study revealed indeed that this trade-off was already visible at early stage through an earlier cessation of tiller production due to *qTSN* introgression, which coincided with the initiation at meristem level of phytomers with potentially larger leaves and internodes. Although it cannot be concluded if this early effect impacts directly tillering or organ dimensioning at meristem level, it seems worth going deeper in the understanding of the physiological regulation of this allele on plant functioning including contrasted cropping condition like limited radiation (i.e., during wet season in the tropics or N availability) in further studies.

## Author contributions

TL, SY, MD, and DL participated in the design of the study; DA, AD participated in performing the research; AF and AC participated in biochemistry analysis; DA, TL, and DL participated in data analysis and wrote the manuscript. All authors read and approved the final manuscript.

### Conflict of interest statement

The authors declare that the research was conducted in the absence of any commercial or financial relationships that could be construed as a potential conflict of interest.

## Supplementary material

Figure S1 **The relative (NIL-P) of IRRI146 background in GH-CNRS under control**. Leaf number on the main tiller **(A)** Tiller number per plant **(B)** Stem length of the main tiller **(C)** Individual leaf area at flowering and spikelet number per panicle at maturity **(D)** Peduncle and internode length at maturity **(E)** Peduncle and internode thickness at maturity **(F)**.

Figure S2 **The relative (NIL1-P) of IR64 background in field-IRRI under low density**. Leaf number on the main tiller **(A)** Tiller number per plant **(B)** Stem length of the main tiller **(C)** Individual leaf area at flowering and spikelet number per panicle at maturity **(D)** Peduncle and internode length at maturity **(E)** Peduncle and internode thickness at maturity **(F)**.

Figure S3 **Relationship between plant shoot growth rate from panicle initiation (PI) to flowering (FLO) and plant grain dry weight at maturity of the parent (black symbol) and the NIL (gray symbol), in IR64 background in GH-CNRS and field trials (A) in IRRI146 background in GH-CNRS and field trials (B) in GH-CNRS in IR64 and IRRI146 backgrounds (C) in field-IRRI experiment in IR64 and IRRI146 backgrounds (D)**. The values are mean ± SE. Regression curves are associated with confidence interval at *P* = 0.05. *n* = 42 for IR64 background and field-IRRI trial, *n* = 34 for IRRI146 background and GH-CNRS trial for regression curve.

Figure S4 **Carbon assimilation (A) and internode starch concentration during panicle development (B) of parent (black), NIL (gray) under isolated and crowded population in GH-IRRI**. The values are mean ± SE. Results of Duncan test for multiple comparisons of each genotype per treatment at 5% level are shown in the letters above the bars. *n* = 3.

## References

[B1] Ashikari M. Sakakibara H. Lin S. Y. Yamamoto T. Takashi T. Nishimura A.. (2005). Cytokinin oxidase regulates rice grain production. Science 309, 741–745. 10.1126/science.1113373 15976269

[B2] Ballaré C. L. Casal J. J. (2000). Light signals perceived by crop and weed plants. Field Crops Res. 67, 149–160. 10.1016/S0378-4290(00)00090-3

[B3] Boehringer S. A. (1984). Methods of Enzymatic Food Analysis using Single Reagents. Mannheim: Boehringer Mannheim GmbH.

[B4] Borràs-Gelonch G. Rebetzke G. J. Richards R. A. Romagosa I. (2012). Genetic control of duration of pre-anthesis phases in wheat (*Triticum aestivum* L.) and relationships to leaf appearance, tillering, and dry matter accumulation. J. Exp. Bot. 63, 69–89. 10.1093/jxb/err230 21920907 PMC3245455

[B5] Brennan J. P. Malabayabas A. (2011). Impacts of IRRI germplasm on the Philippines since 1985, in International Rice Research Institute's Contribution to Rice Varietal Yield Improvement in South-East Asia (Canberra, ACT: Australian Center for International Agricultural Research), 25–43. Impact Assesment Series Report. 74.

[B6] Bueno C. S. Lafarge T. (2009). Higher crop performance of rice hybrids than of elite inbreds in the tropics: 1. hybrids accumulate more biomass during each phenological phase. Field Crops Res. 112, 229–237. 10.1016/j.fcr.2009.03.006

[B7] Chen S. Gao R. Wang H. Wen M. Xiao J. Bian N. (2015). Characterization of a novel reduced height gene (Rht23) regulating panicle morphology and plant architecture in bread wheat. Euphytica 203, 583–594. 10.1007/s10681-014-1275-1

[B8] Chen S. Zeng F. R. Pao Z. Z. Zhang G. P. (2008). Characterization of high-yield performance as affected by genotype and environment in rice. J. Zhejiang Univ. Sci. B 9, 363–370. 10.1631/jzus.B0710603 18500775 PMC2367374

[B9] Dingkuhn M. Laza M. R. C. Kumar U. Mendez K. S. Collard B. Jagadish K. (2015). Improving yield potential of tropical rice: achieved levels and perspectives through improved ideotypes. Field Crops Res. 182, 43–59. 10.1016/j.fcr.2015.05.025

[B10] Dong X. Wang X. Zhang L. Yang Z. Xin X. Wu S.. (2013). Identification and characterization of *OsEBS*, a gene involved in enhanced plant biomass and spikelet number in rice. Plant Biotechnol. J. 11, 1044–1057. 10.1111/pbi.12097 23924074

[B11] Dong Y. J. Kamiunten H. Ogawa T. Tsuzuki E. Terao H. Lin D. Z. (2004). Mapping of QTLs for leaf developmental behavior in rice (*Oryza sativa* L.). Euphytica 138, 169–175. 10.1023/B:EUPH.0000046799.21410.13

[B12] Dreccer M. F. Chapman S. C. Ogbonnaya F. C. Borgognone M. G. Trethowan R. M. (2008). Crop and environmental attributes underpinning genotype by environment interaction in synthetic-derived bread wheat evaluated in Mexico and Australia. Aust. J. Agric. Res. 59, 447–460. 10.1071/AR07220

[B13] Fujita D. Ebron L. A. Araki E. Kato H. Khush G. S. Sheehy J. E.. (2010). Fine mapping of a gene for low-tiller number, Ltn, in japonica rice (*Oryza sativa* L.) variety Aikawa 1. Theor. Appl. Genet. 120, 1233–1240. 10.1007/s00122-009-1251-7 20062964

[B14] Fujita D. Santos R. E. Ebron L. A. Telebanco-Yanoria M. J. Kato H. Kobayashi S. (2009). Development of introgression lines of an Indica-type rice variety, IR64, for unique agronomic traits and detection of the responsible chromosomal regions. Field Crops Res. 114, 244–254. 10.1016/j.fcr.2009.08.004

[B15] Fujita D. Tagle A. G. Ebron L. A. Fukuta Y. Kobayashi N. (2012). Characterization of near-isogenic lines carrying QTL for high spikelet number with the genetic background of an indica rice variety IR64 (*Oryza sativa* L.). Breed. Sci. 62, 18–26. 10.1270/jsbbs.62.18 23136510 PMC3405953

[B16] Fujita D. Trijatmiko K. R. Tagle A. G. Sapasap M. V. Koide Y. Sasaki K.. (2013). *NAL1* allele from a rice landrace greatly increases yield in modern *indica* cultivars. Proc. Natl. Acad. Sci. U.S.A. 110, 20431–20436. 10.1073/pnas.1310790110 24297875 PMC3870739

[B17] Fujita K. Yoshida S. (1984). Partitioning of photosynthates between panicle and vegetative organs of rice under different planting densities. Soil Sci. Plant Nutr. 30, 519–525. 10.1080/00380768.1984.10434719

[B18] Gaju O. Reynolds M. P. Sparkes D. L. Mayes S. Ribas-Vargas G. Crossa J. (2014). Relationships between physiological traits, grain number and yield potential in a wheat DH population of large spike phenotype. Field Crops Res. 164, 126–135. 10.1016/j.fcr.2014.05.015

[B19] Hashida Y. Aoki N. Kawanishi H. Okamura M. Ebitani T. Hirose T. (2013). A near isogenic line of rice carrying chromosome segments containing OsSPS1 of Kasalath in the genetic background of Koshihikari produces an increased spikelet number per panicle. Field Crops Res. 149, 56–62. 10.1016/j.fcr.2013.04.020

[B20] Huang X. Qian Q. Liu Z. Sun H. He S. Luo D.. (2009). Natural variation at the DEP1 locus enhances grain yield in rice. Nat. Genet. 41, 494–497. 10.1038/ng.352 19305410

[B21] Ikeda K. Ito M. Nagasawa M. Kyozuka J. Nagato Y. (2007). Rice *ABERRANT PANICLE ORGANIZATION* 1, encoding an F-box protein, regulates meristem fate. Plant J. 51, 1030–1040. 10.1111/j.1365-313X.2007.03200.x 17666027

[B22] Itoh Y. Shimizu H. (2012). Phyllochron dynamics during the course of late shoot development might be affected by reproductive development in rice (*Oryza sativa* L.). Dev. Genes Evol. 222, 341–350. 10.1007/s00427-012-0419-3 23096942

[B23] Jamil M. Charnikhova T. Houshyani B. van Ast A. Bouwmeester H. (2012). Genetic variation in strigolactone production and tillering in rice and its effect on *Striga hermonthica* infection. Planta 235, 473–484. 10.1007/s00425-011-1520-y 21947621 PMC3288373

[B24] Khush G. S. (1987). Rice breeding: past, present and future. J. Genet. 66, 195–216. 10.1007/BF02927713

[B25] Komatsu K. Maekawa M. Ujiie S. Satake Y. Furutani I. Okamoto H.. (2003). LAX and SPA: major regulators of shoot branching in rice. Proc. Natl. Acad. Sci. U.S.A. 100, 11765–11770. 10.1073/pnas.1932414100 13130077 PMC208832

[B26] Lafarge T. A. Broad I. J. Hammer G. L. (2002). Tillering in grain sorghum over a wide range of population densities: identification of a common hierarchy for tiller emergence, leaf area development and fertility. Ann. Bot. 90, 87–98. 10.1093/aob/mcf152 12125776 PMC4233856

[B27] Lafarge T. Bueno C. S. (2009). Higher crop performance of rice hybrids than of elite inbreds in the tropics: 2. does sink regulation, rather than sink size, play a major role? Field Crops Res. 114, 434–440. 10.1016/j.fcr.2009.03.007

[B28] Lafarge T. Seassau C. Martin M. Bueno C. Clément-Vidal A. Shreck E. (2010). Regulation and recovery of sink strength in rice plants grown under changes in light intensity. Funct. Plant Biol. 37, 413–428. 10.1071/FP09137

[B29] Li X. Qian Q. Fu Z. Wang Y. Xiong G. Zeng D.. (2003). Control of tillering in rice. Nature 422, 618–621. 10.1038/nature01518 12687001

[B30] Liu G. L. Mei H. W. Yu X. Q. Zou G. H. Liu H. Y. Hu S. P. (2008). QTL analysis of panicle neck diameter, a trait highly correlated with panicle size, under well-watered and drought conditions in rice (*Oryza sativa* L.). Plant Sci. 174, 71–77. 10.1016/j.plantsci.2007.09.011

[B31] Liu W. Zhang D. Tang M. Li D. Zhu Y. Zhu L.. (2013). THIS1 is a putative lipase that regulates tillering, plant height, and spikelet fertility in rice. J. Exp. Bot. 64, 4389–4402. 10.1093/jxb/ert256 24085578

[B32] Liu Y. Wang Q. Ding Y. Li G. Xu J. Wang S. (2011). Effects of external ABA, GA3 and NAA on the tiller bud outgrowth of rice is related to changes in endogenous hormones. Plant Growth Regul. 65, 247–254. 10.1007/s10725-011-9594-x

[B33] Miura K. Ikeda M. Matsubara A. Song X. J. Ito M. Asano K.. (2010). *OsSPL14* promotes panicle branching and higher grain productivity in rice. Nat. Genet. 42, 545–592. 10.1038/ng.592 20495564

[B34] Nemoto K. Morita S. Baba T. (1995). Shoot and root development in rice related to the phyllochron. Crop Sci. 35, 24–29. 10.2135/cropsci1995.0011183X003500010005x

[B35] Okami M. Kato Y. Kobayashi N. Yamagishi J. (2014). Agronomic performance of an IR64 introgression line with large leaves derived from New Plant Type rice in aerobic culture. Eur. J. Agron. 58, 11–17. 10.1016/j.eja.2014.03.001

[B36] Okami M. Kato Y. Kobayashi N. Yamagishi J. (2015). Morphological traits associated with vegetative growth of rice (*Oryza sativa* L.) during the recovery phase after early-season drought. Eur. J. Agron. 64, 58–66. 10.1016/j.eja.2014.12.006

[B37] Okami M. Kato Y. Yamagishi J. (2012). Allometric relationship between the size and number of shoots as a determinant of adaptations in rice to water-saving aerobic culture. Field Crops Res. 131, 17–25. 10.1016/j.fcr.2012.02.014

[B38] Okawa S. Makino A. Mae T. (2003). Effect of irradiance on the partitioning of assimilated carbon during the early phase of grain filling in rice. Ann. Bot. 92, 357–364. 10.1093/aob/mcg147 12853283 PMC4257509

[B39] Qi J. Qian Q. Bu Q. Li S. Chen Q. Sun J.. (2008). Mutation of the rice *narrow leaf1* gene, which encodes a novel protein, affects vein patterning and polar auxin transport. Plant Physiol. 147, 1947–1959. 10.1104/pp.108.118778 18562767 PMC2492643

[B40] Rebolledo M. C. Dingkuhn M. Clément-Vidal A. Rouan L. Luquet D. (2012). Phenomics of rice early vigour and drought response: are sugar related and morphogenetic traits relevant? Rice 5:22. 10.1186/1939-8433-5-22 24279832 PMC4883731

[B41] Streck N. A. Lago I. Bosco L. C. de Paula G. M. Oliveira F. B. Gabriel L. F. (2009). Relationship between panicle differentiation and main stem leaf number in rice genotypes and red rice biotypes. Sci. Agric. 66, 195–203. 10.1590/S0103-90162009000200008

[B42] Terao T. Nagata K. Morino K. Hirose T. (2010). A gene controlling the number of primary rachis branches also controls the vascular bundle formation and hence is responsible to increase the harvest index and grain yield in rice. Theor. Appl. Genet. 120, 875–893. 10.1007/s00122-009-1218-8 20151298

[B43] Tivet F. Pinheiro B. D. S. Raissac M. D. Dingkuhn M. (2001). Leaf blade dimensions of rice (*Oryza sativa* L. and *Oryza glaberrima Steud.)*. Relationships between tillers and the main stem. Ann. Bot. 88, 507–511. 10.1006/anbo.2001.1447

[B44] Ugarte C. C. Trupkin S. A. Ghiglione H. Slafer G. Casal J. J. (2010). Low red/far-red ratios delay spike and stem growth in wheat. J. Exp. Bot. 61, 3151–3162. 10.1093/jxb/erq140 20497971 PMC2892155

[B45] Wang W. Li G. Zhao J. Chu H. Lin W. Zhang D.. (2014). DWARF TILLER1, a WUSCHEL-related homeobox transcription factor, is required for tiller growth in rice. PLoS Genet. 10:e1004154. 10.1371/journal.pgen.1004154 24625559 PMC3952828

[B46] Weng X. Wang L. Wang J. Hu Y. Du H. Xu C.. (2014). Grain number, plant height, and heading date7 is a central regulator of growth, development, and stress response. Plant Physiol. 164, 735–747. 10.1104/pp.113.231308 24390391 PMC3912102

[B47] Wu L. L. Liu Z. L. Wang J. M. Zhou C. Y. Chen K. M. (2011). Morphological, anatomical, and physiological characteristics involved in development of the large culm trait in rice. Aust. J. Crop Sci. 5, 1356–1363.

[B48] Zhang B. Yamagishi J. (2010). Response of spikelet number per panicle in rice cultivars to three transplanting densities. Plant Prod. Sci. 13, 279–288. 10.1626/pps.13.279

