# Supplementary figures and images for: The qTSN Positive Effect on Panicle and Flag Leaf Size of Rice is Associated with an Early Down-Regulation of Tillering
Source: Front Plant Sci. 2016 Jan 7;6:1197. doi: 10.3389/fpls.2015.01197 (PMC4703761; doi:10.3389/fpls.2015.01197)

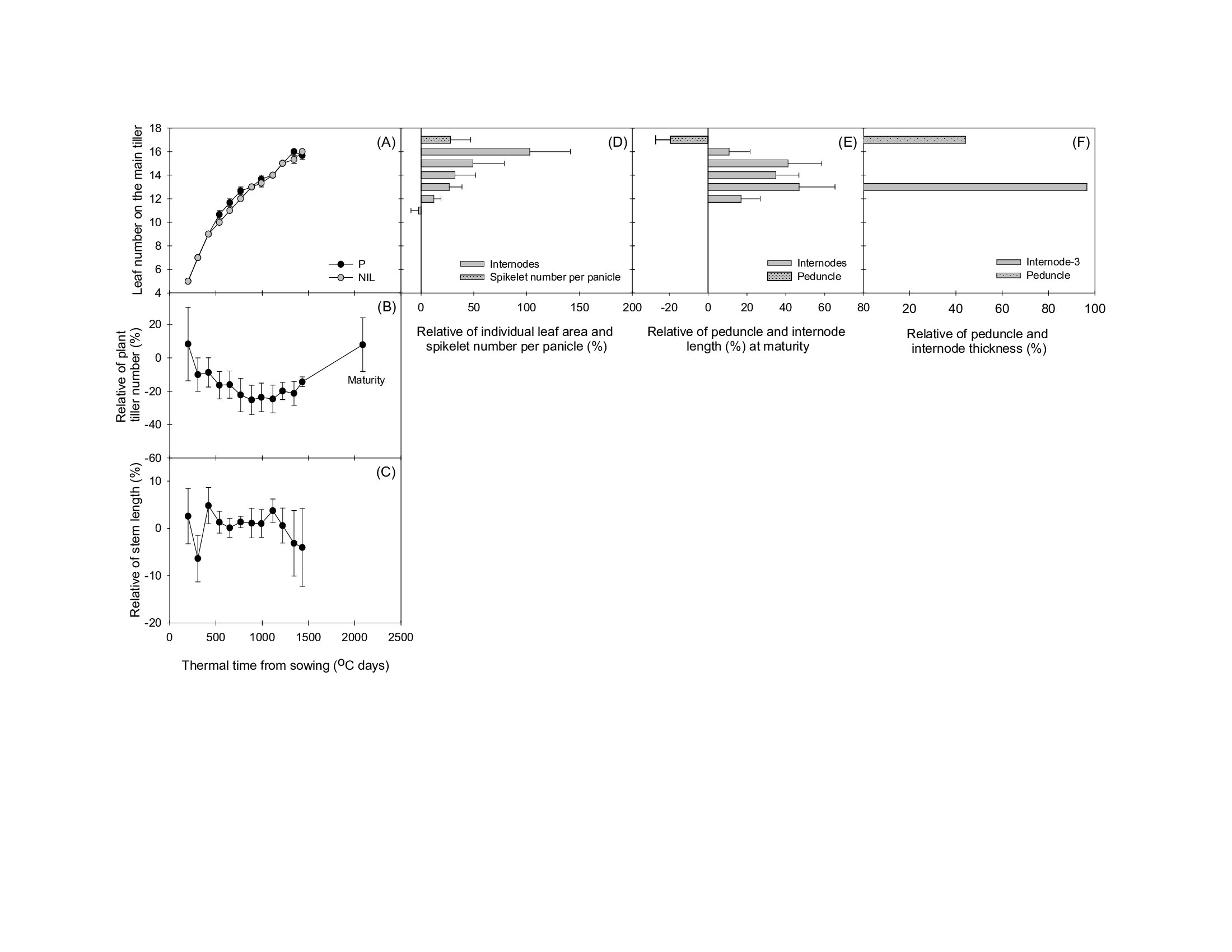

Supplement: Figure S1 — The relative (NIL-P) of IRRI146 background in GH-CNRS under control. Leaf number on the main tiller (A) Tiller number per plant (B) Stem length of the main tiller (C) Individual leaf area at flowering and spikelet number per panicle at maturity (D) Peduncle and internode length at maturity (E) Peduncle and internode thickness at maturity (F). [file Image1.JPEG]

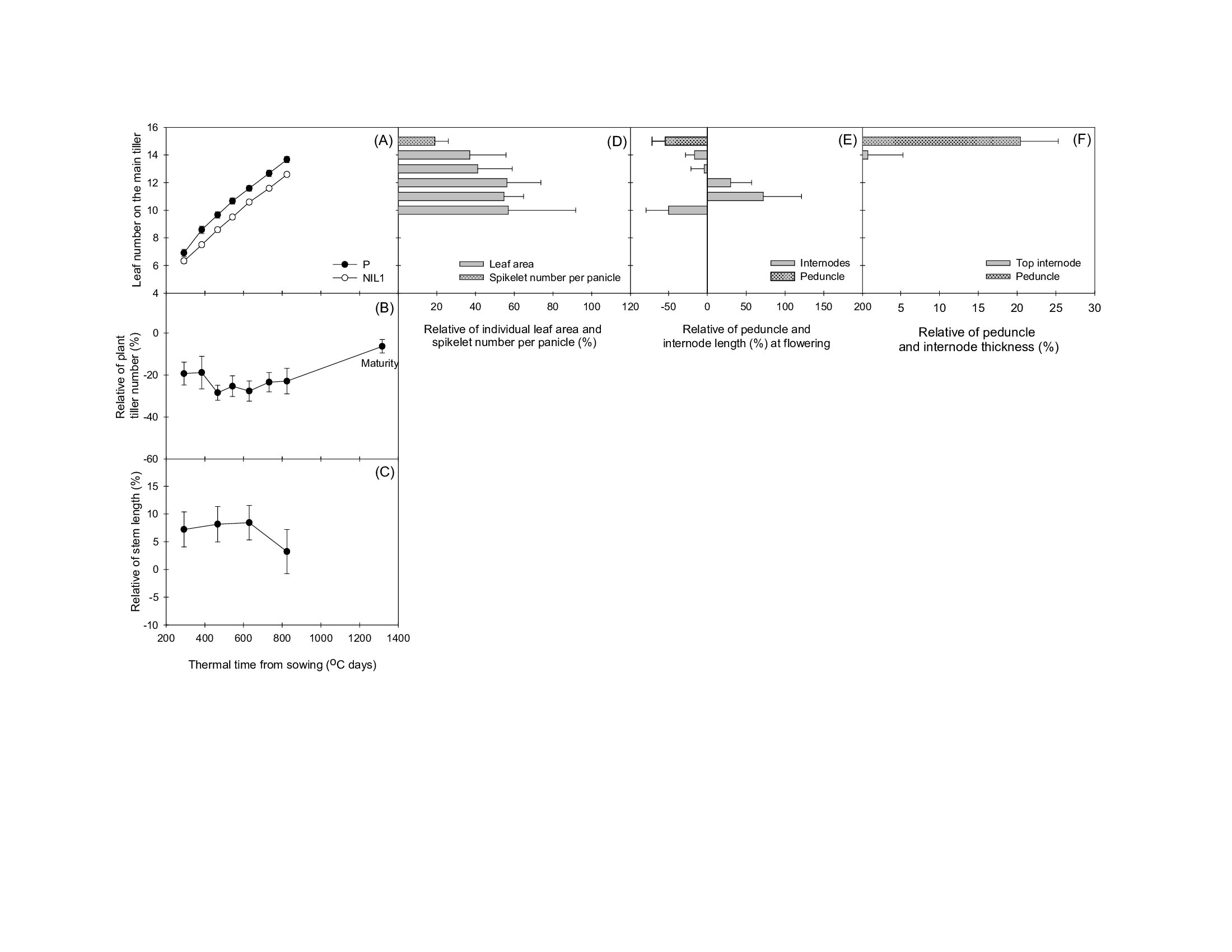

Supplement: Figure S2 — The relative (NIL1-P) of IR64 background in field-IRRI under low density. Leaf number on the main tiller (A) Tiller number per plant (B) Stem length of the main tiller (C) Individual leaf area at flowering and spikelet number per panicle at maturity (D) Peduncle and internode length at maturity (E) Peduncle and internode thickness at maturity (F). [file Image2.JPEG]

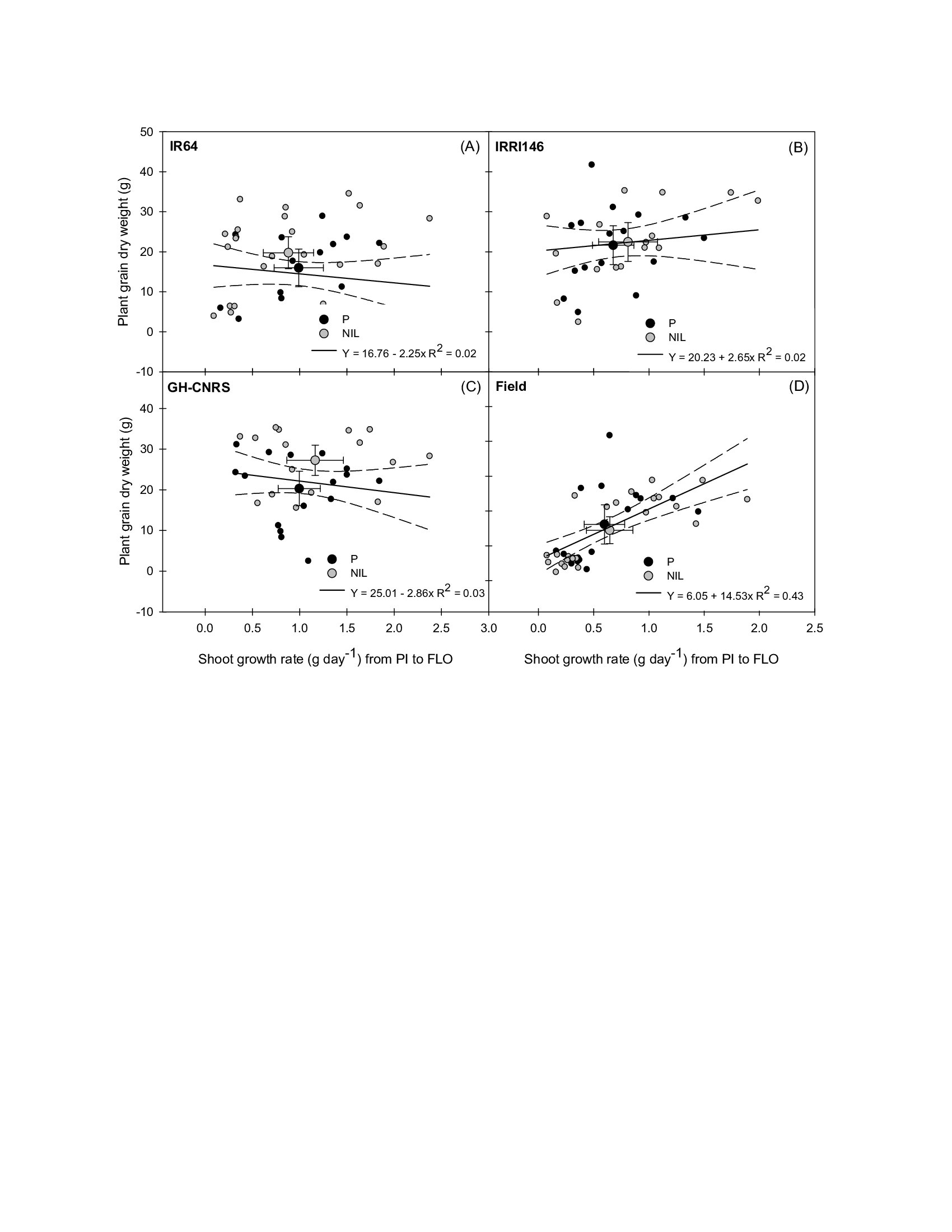

Supplement: Figure S3 — Relationship between plant shoot growth rate from panicle initiation (PI) to flowering (FLO) and plant grain dry weight at maturity of the parent (black symbol) and the NIL (gray symbol), in IR64 background in GH-CNRS and field trials (A) in IRRI146 background in GH-CNRS and field trials (B) in GH-CNRS in IR64 and IRRI146 backgrounds (C) in field-IRRI experiment in IR64 and IRRI146 backgrounds (D). The values are mean ± SE. Regression curves are associated with confidence interval at P = 0.05. n = 42 for IR64 background and field-IRRI trial, n = 34 for IRRI146 background and GH-CNRS trial for regression curve. [file Image3.JPEG]

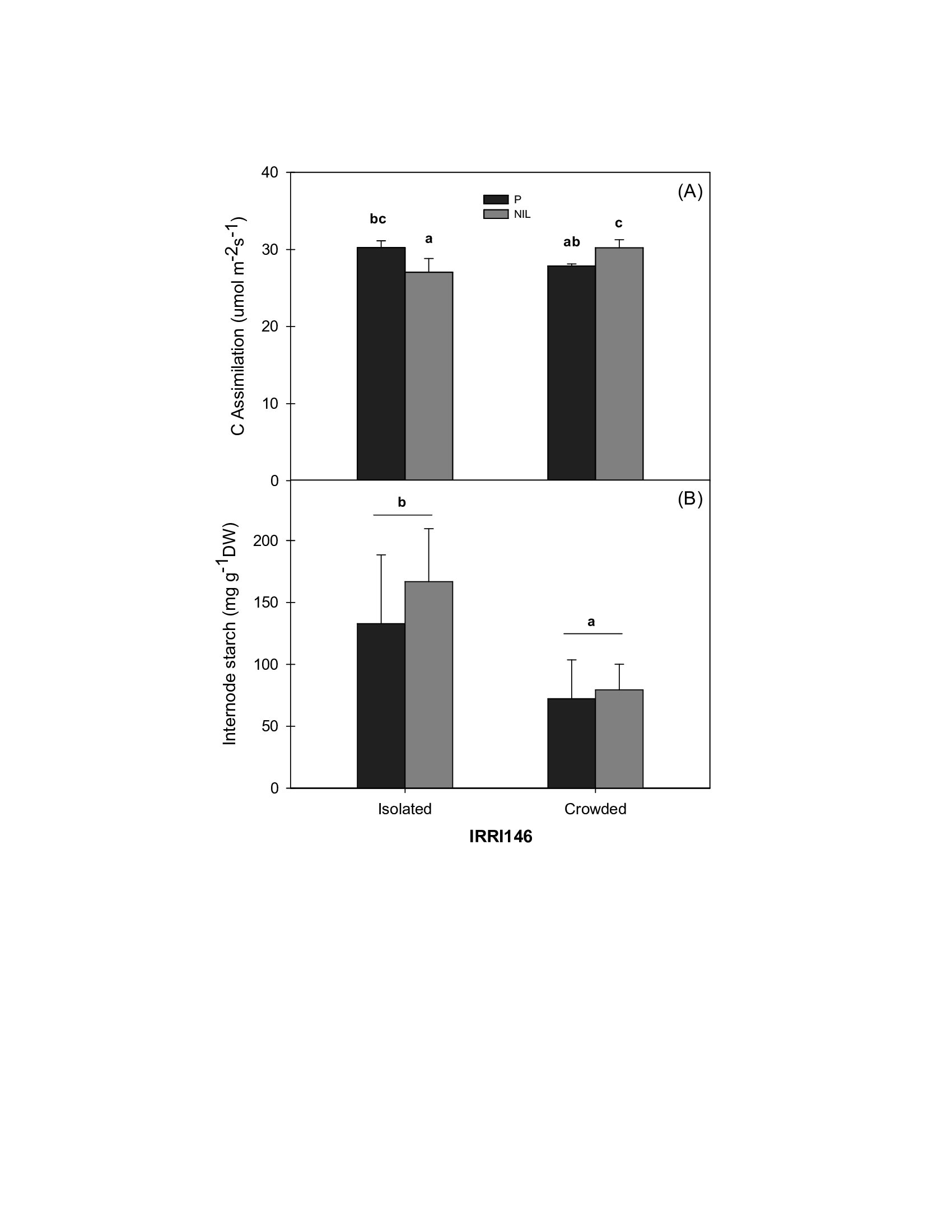

Supplement: Figure S4 — Carbon assimilation (A) and internode starch concentration during panicle development (B) of parent (black), NIL (gray) under isolated and crowded population in GH-IRRI. The values are mean ± SE. Results of Duncan test for multiple comparisons of each genotype per treatment at 5% level are shown in the letters above the bars. n = 3. [file Image4.JPEG]
